# In‐Wheel Piezoelectric DC Power Generator With Zero Resistive Torque

**DOI:** 10.1002/advs.202522932

**Published:** 2026-01-28

**Authors:** Hyun Soo Kim, Hyunseok Song, In Woo Oh, Dong‐Gyu Lee, Tae Kyoung Yoon, Jeyeon Lee, Byoung Jin Yoon, So‐Min Song, Iman M. Imani, Seohyun Cho, Seong Jin Kim, Chong‐Yun Kang, Sahn Nahm, Yong Seok Park, Kyung‐Hoon Cho, Jungho Ryu, Jeong Min Baik, Jun Chen, Sunghoon Hur, Hyun‐Cheol Song

**Affiliations:** ^1^ Electronic and Hybrid Material Research Center Korea Institute of Science and Technology (KIST) Seoul Republic of Korea; ^2^ Department of Bioengineering University of California, Los Angeles Los Angeles California USA; ^3^ Department of Material Science and Engineering Korea University Seoul Republic of Korea; ^4^ KU‐KIST Graduate School of Converging Science and Technology Korea University Seoul Republic of Korea; ^5^ KIST‐SKKU Carbon‐Neutral Research Center Sungkyunkwan University (SKKU) Suwon Republic of Korea; ^6^ Department of Micro/Nano Systems Korea University Seoul Republic of Korea; ^7^ Department of Mechanical Engineering Korea University Seoul Republic of Korea; ^8^ Extreme Materials Research Center Korea Institute of Science and Technology (KIST) Seoul Republic of Korea; ^9^ Department of Mechanical Engineering California State University Fullerton California USA; ^10^ School of Materials Science and Engineering Kumoh National institute of Technology Gumi Republic of Korea; ^11^ School of Materials Science and Engineering Yeungnam University Gyeongsan Republic of Korea; ^12^ School of Advanced Materials and Engineering Sungkyunkwan University (SKKU) Suwon Republic of Korea

**Keywords:** DC generator, Internet of Things, piezoelectric, PMN‐PT, self‐power

## Abstract

Conventional electromagnetic generators are limited to low‐power applications owing to their mechanical complexity, bulky design, and reduced efficiency at small scales. Their performance is further hindered by the resistive torque according to Lenz's law, which makes them unsuitable for low‐load environments. To overcome these challenges, we propose a novel in‐wheel direct‐current piezoelectric generator (DC‐PG), which enables efficient power generation with zero resistive torque and does not require rectification. The system leverages continuous in‐phase polarization during rotation to deliver a stable low‐ripple DC output with minimal energy loss. Integrated into a suitcase wheel without adding weight or volume, the DC‐PG achieved a peak power of 4.28 mW under realistic conditions (20 kg load, 3–5 km/h), surpassing conventional AC‐based piezoelectric systems. It successfully powered a wireless Internet of things (IoT) location sensor, charging a 5 V capacitor in 134 s. This compact rectification‐free generator is a viable solution for small‐scale power generation and self‐powered IoT applications. Its high‐efficiency performance and elimination of electromagnetic resistance open new possibilities for energy autonomy in low‐speed, low‐load environments.

## Graphical abstract

In this paper, we present a wheel‐shaped direct‐current piezoelectric generator (DC‐PG) that fundamentally overcomes these constraints by producing phase‐stable DC electricity directly from rolling motion. By embedding this system into suitcase wheels, we demonstrated a fully integrated real‐time location tracking system powered solely by mechanical motion, eliminating the need for external power or batteries.

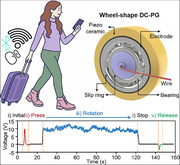

## 1 Introduction

Since Michael Faraday's seminal discovery of electromagnetic induction in the 19th century, electromagnetic generators have played a pivotal role in shaping modern industry and everyday life, forming the backbone of large‐scale centralized power systems [1]. While traditional electromagnetic generators effectively deliver high power outputs suitable for wide‐area distribution, they face considerable limitations when scaled down to satisfy the compact and distributed energy requirements prevalent in contemporary mobile electronics and portable devices. Devices, such as smartphones, remote sensors, and wearable electronics, require compact, efficient, and reliable on‐site power generation solutions [2, 3, 4, 5, 6, 7, 8, 9, 10, 11]. However, electromagnetic generators inherently suffer from high torque requirements imposed by Lenz's law, resulting in significant mechanical complexity and diminishing efficiency at smaller scales. Moreover, the alternating current (AC) produced by these systems necessitates further rectification and direct current (DC)‐DC conversion steps to match the DC requirements of most modern electronics, incurring additional energy losses and increasing system complexity and size [12, 13, 14, 15, 16, 17, 18, 19, 20].

The underlying issue arises from the electromagnetic generation principles: increasing the output current induces magnetic fields opposing the generator's rotation, thus demanding a higher input torque to sustain rotational motion. Consequently, the efficiency dramatically decreases at smaller scales owing to magnetic drag and the complex mechanical solutions required to counteract it. Further efficiency losses occur through AC‐DC conversion and subsequent voltage regulation, exacerbating the system inefficiencies and complexity. As end‐use devices typically operate on DC power, generating electricity in proximity to such devices eliminates the need for AC generation and avoids the energy losses associated with AC‐DC conversion. This is even more problematic for small electronic devices that rely on battery recharging.

To address these challenges, we introduce an innovative piezoelectric‐based energy generation technology designed to generate a DC output without the torque resistance inherent in electromagnetic systems [21]. This approach leverages the intrinsic properties of piezoelectric materials, which directly convert mechanical stress into electrical energy, thereby effectively bypassing the magnetic braking effects imposed by Lenz's law [22, 23]. This fundamental advantage enables significantly reduced mechanical input requirements for operation and achieves efficient and stable power generation, even at compact scales. Furthermore, direct DC generation from the piezoelectric mechanism eliminates rectification losses and provides immediate and usable power with minimal additional conditioning for battery charging and device operation.

In this paper, we present a novel DC power generation mechanism that encompasses the material development, device design, and implementation of a piezoelectric DC generator. Our design emphasizes capability of the generator to deliver a stable, high‐quality DC output while significantly minimizing both mechanical and electrical inefficiencies. By integrating energy harvesting with a direct DC output in a compact, torque‐free system, this approach represents a substantial advancement in lightweight, efficient, robust, and portable power solutions. This directly addresses the growing demand for decentralized, device‐centric energy applications, thereby facilitating their widespread adoption in Internet of things (IoT) systems, small‐scale power generation, and other portable technologies.

## 2 Results and Discussion

**FIGURE 1:**
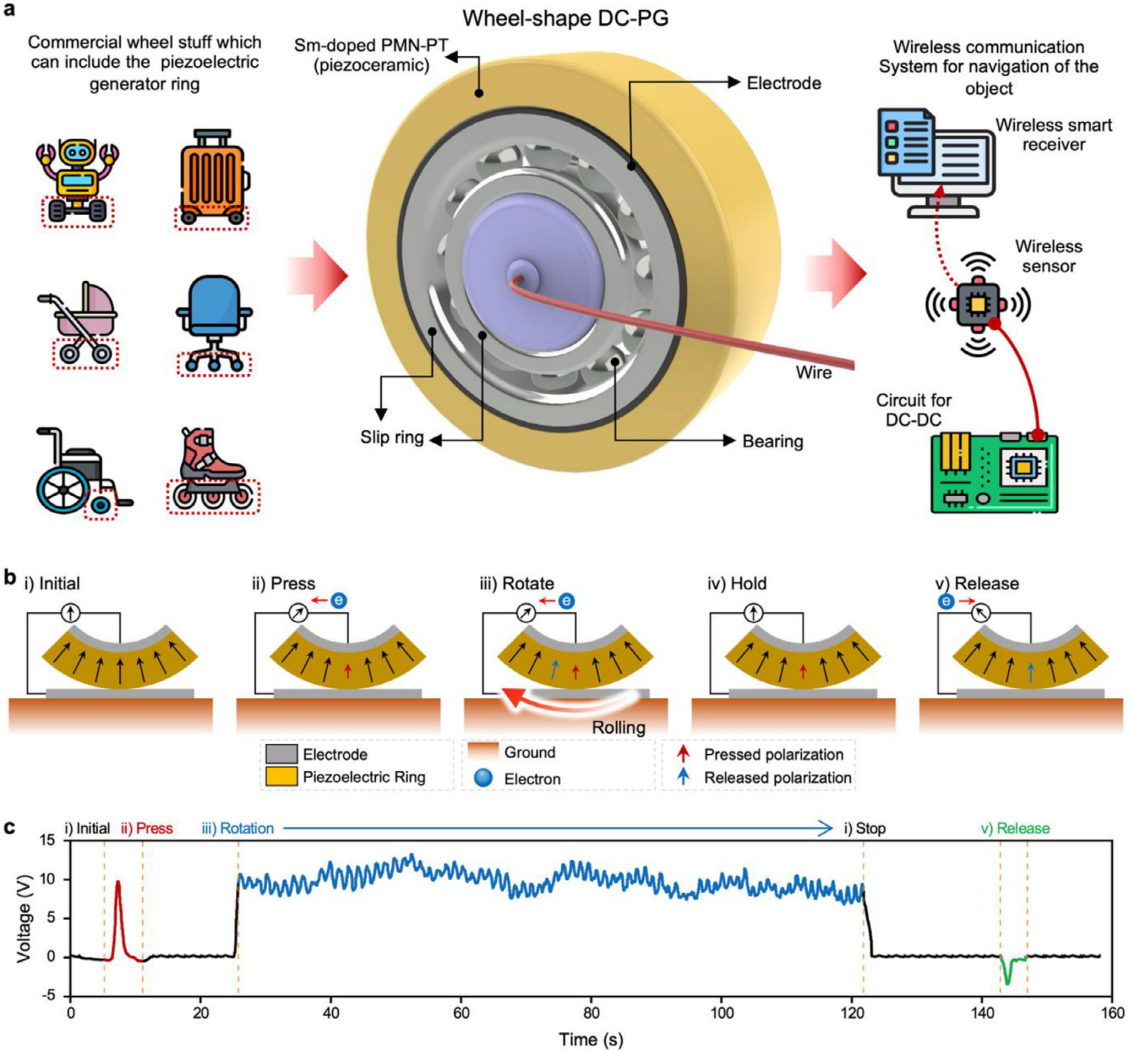
Conceptual design and working principle of the in‐wheel DC‐PG. (a) Conceptual illustration of an energy‐harvesting system using an in‐wheel DC‐PG embedded in a moving vehicle. (b) Schematic of the DC generation mechanism. Compressive pressure applied during rotation induces internal polarization in the piezoelectric material, resulting in potential development across the electrodes. Only compressive polarization contributes to the net output, whereas tensile polarization, released upon separation, does not. Continuous rotation selectively accumulates in‐phase compressive polarization, enabling sustained DC generation. The symmetry of this mechanism ensures consistent output polarity regardless of the rotation direction. (c) Experimentally measured open‐circuit voltage profile validating the DC generation process. The voltage output matches the expected behavior based on polarization dynamics.

The fundamental operating principle and schematic of our wheel‐shaped DC piezoelectric generator (DC‐PG) is illustrated in Figure 1. By integrating this specially engineered piezoelectric generator into a wheel structure, we achieved efficient DC generation without additional bulk or weight, thus providing significant advantages for powering compact electronic systems such as IoT sensors. Unlike conventional electromagnetic generators, which inherently produce mechanical resistance owing to the magnetic drag induced by Lenz's law, where the large flux changes required for higher power generation inevitably result in substantial torque demands, our DC‐PG operates free of such limitations. Specifically, conventional electromagnetic systems require significant mechanical torque to overcome internal coil and permanent magnet interactions, making them suitable for large‐scale applications such as modern wind turbines, but they are increasingly inefficient when scaled down. In contrast, the resistance of the DC‐PG is limited solely to the minimal rolling friction, which is a negligible force independent of the rotational speed, enabling efficient operation with a significantly lower torque. This characteristic significantly facilitates miniaturization, rendering our DC‐PG highly advantageous for small‐scale applications.

Figure 1a shows a schematic of the DC‐PG device configuration. The generator consists of a ring‐shaped piezoelectric material mounted securely on a bearing that enables stable rotational motion. A ring‐shaped piezoelectric material was radially poled and the electrode on the outer surface was subsequently removed to enable DC generation. Additionally, a slip ring mechanism was incorporated to eliminate wire entanglement issues typically encountered during continuous rotation, as shown in Figure S1.

Figure 1b illustrates the operating principle of DC generation, demonstrating how the mechanical energy from wheel rotation and continuous pressure variation is efficiently converted into electrical energy. When mechanical pressure is applied to the wheel‐shaped piezoelectric material, the magnitude of the internal polarization changes, generating an electric current via the piezoelectric effect (Figure 1b(ii), ‘Press’). As the wheel rotates, compressed segments of the piezoelectric material move away from the pressure point, and previously uncompressed regions come into contact with the pressure source, thereby continuously applying mechanical stress to new areas (Figure 1b(iii), ‘Rotate’). A crucial aspect of this mechanism is that only compressive polarization contributes to the generation of electric potential. By contrast, the polarization resulting from the release of pressure does not contribute to current generation, because the corresponding segment is immediately detached from the bottom electrode upon decompression, preventing electrical potential cancellation. Consequently, electrical potential is primarily generated during the compression phase. This cycle of compressive polarization is repeated through continuous rotation under sustained pressure, enabling the stable production of DC electricity. Notably, when pressure is removed without rotation, a negative voltage is generated owing to the release‐induced (extensive) polarization, as shown in Figure 1b(v), ‘Release.’ This negative signal strongly supports this idea that the DC generation mechanism is fundamentally governed by the piezoelectric effect.

Conventional piezoelectric generators operate based on cyclic mechanical deformation, in which the electrical output arises only during the dynamic transitions between pressing and releasing. This inherently pulsed operation leads to sharp voltage spikes and AC characteristics, requiring external rectification to obtain a usable DC. Even with rectification, the resulting RMS voltage remains low, and substantial energy is lost during the conversion process. To address this issue, first‐generation DC‐PGs were developed by incorporating semiconducting materials that form internal p–n junctions (Figure S2). These devices selectively allow current to flow in one direction, acting as self‐rectifying systems without external circuits. However, they fundamentally remain pulse‐based devices, as the electrical output still arises only during momentary changes in mechanical stress. This results in high peak voltages but low RMS values and energy densities because of the limited duty cycle of the effective generation and internal leakage losses [24, 25, 26, 27, 28, 29, 30, 31, 32, 33]. In contrast, the second‐generation DC‐PG introduced in this study functions in a fundamentally different way. Instead of generating electricity only during brief moments of pressing and releasing, the device remains under constant compression while rotating. As the wheel turns, the mechanical stress moves smoothly across the piezoelectric material, causing the polarization to change continuously. This steady and directional change in polarization results in a continuous flow of DC, rather than intermittent pulses. Crucially, the generated signal is not a sequence of pulses but a genuine DC with a high RMS voltage (*V_RMS_*; 10.45 V) and a low crest factor (*V_Max_*/*V_RMS_*: 1.27), approaching unity. The calculated crest factor of 1.27 is significantly lower than that of typical piezoelectric AC generators (crest factor ≈ 2–4), confirming that our device produces a low‐fluctuation DC output rather than sharp pulsed signals. This results in a markedly higher effective energy density and eliminates the need for rectification or charge balancing. The output is inherently stable and efficient, and power can be delivered directly to the electronic systems without additional circuitry. This key distinction establishes our second‐generation DC‐PG as a truly continuous and high‐performance solution, beyond simply suppressing the negative portion of the AC waveform.

Figure 1c shows the experimentally measured voltage output of the wheel‐shaped DC‐PG under realistic rolling conditions. Frequency‐domain analysis (Fast Fourier Transform, FFT) result demonstrates that the signal contains no significant alternating components, and its spectral energy is concentrated near 0–1 Hz, corresponding to the slow fluctuation envelope of the rolling motion rather than AC oscillations. This further verifies that the generated voltage is fundamentally DC in nature. (Figure S3) The empirical results demonstrate the exceptional stability and reliability of the generated DC voltage, thereby affirming the practical efficacy of the proposed approach. The high efficiency of electrical energy conversion was attributed to the strategic integration of advanced piezoelectric material properties with a carefully optimized device structure.

### 2.1 Theoretical Basis of DC Output in Wheel‐Shaped Piezo‐Generator

The DC generation mechanism of the wheel‐shaped DC‐PG fundamentally differs from that of conventional piezoelectric generators, particularly in terms of the application of mechanical stress and the harvesting of electrical output. Traditional piezoelectric generators operate based on periodic pressing and released motions that induce alternating polarization changes and result in high‐voltage pulse trains or AC outputs. These systems rely on transient variations in pressure over time to generate electrical charges, necessitating subsequent rectification to produce usable DC for modern electronics. This inherently limits their energy density and practical efficiency owing to both electrical losses and mechanical complexity.

By contrast, the DC‐PG introduced herein was designed to maintain a constant mechanical pressure while continuously rotating. Rather than varying the pressure temporally, the DC‐PG utilizes the spatial modulation of the active piezoelectric area. As the wheel rotates, new regions of the piezoelectric material sequentially come into contact with the compressive load, whereas the previously stressed regions move away from the surface electrode. This motion results in a continuous sequence of polarization changes across the material, as different regions are sequentially subjected to and released from mechanical stress, even though the pressure magnitude remains constant. As the polarization vector within each segment of the material is altered as it enters and exits the stress zone, a charge is continuously generated, leading to a sustained DC output.

This spatially progressive activation of the piezoelectric domains enables the generator to produce charges without requiring abrupt changes in the applied force. The rotational motion transforms the linear displacement into a time‐varying activation of the piezoelectric surface, thereby creating a continuous current flow. Importantly, as the applied mechanical stress does not oscillate between compression and release, the resulting electrical signal remains unidirectional and inherently DC.

Another advantage of this mechanism is its exceptionally low mechanical resistance. Unlike electromagnetic generators, which experience torque drag owing to Lenz's law, the DC‐PG encounters minimal rolling friction as it operates. This enables for efficient energy harvesting at a low torque input, making the system highly suitable for miniaturized applications such as wearable devices, IoT sensors, and autonomous mobile systems. Furthermore, as the output is natively DC, the system avoids the energy losses associated with external rectification circuits and AC‐DC conversion stages. A more detailed theoretical derivation of the governing equations, including the voltage, current, and internal resistance, is provided in Note S1.

### 2.2 Design of High‐Figure‐of‐Merit Piezoelectric Ceramics for DC‐PG

**FIGURE 2:**
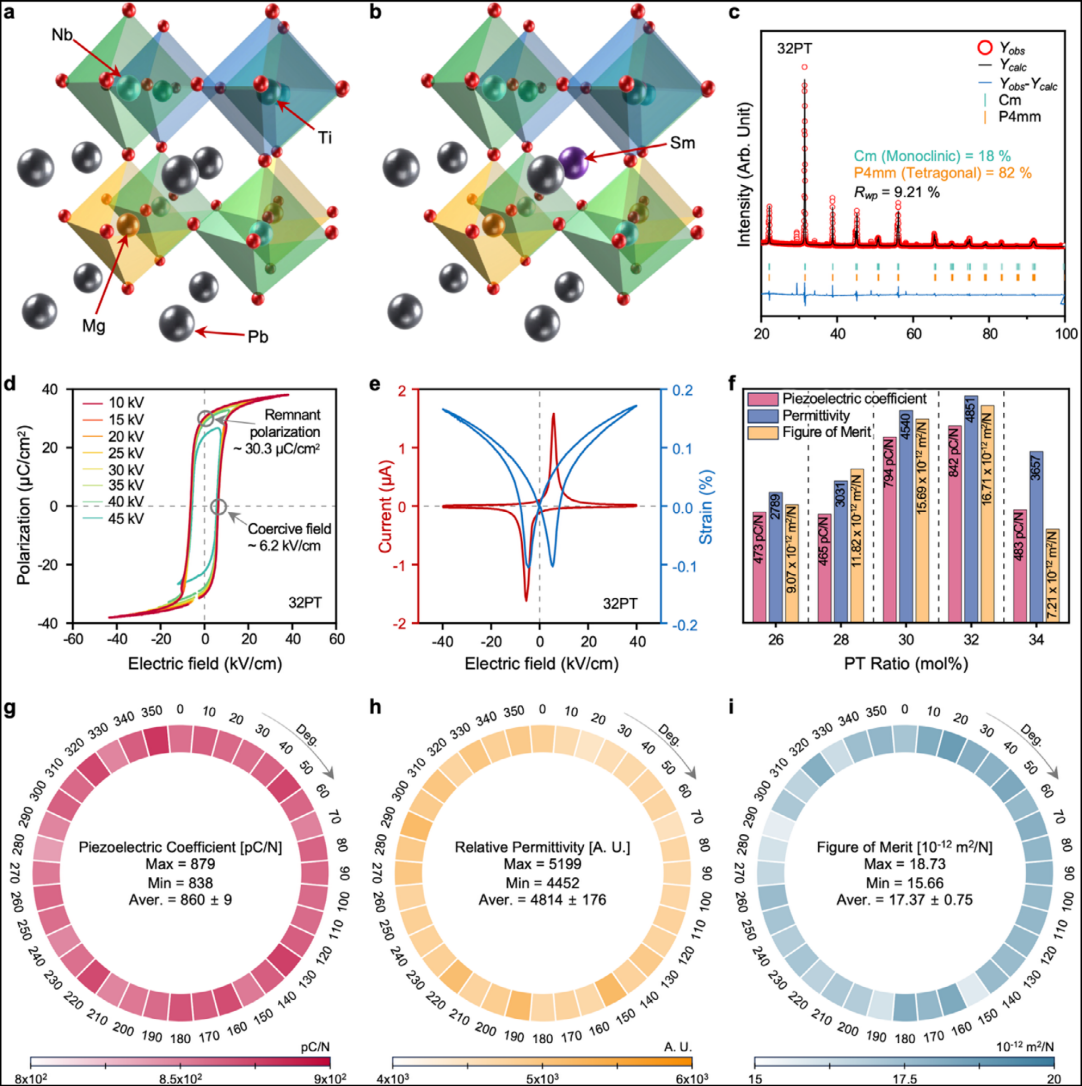
Design of DC piezoelectric generating materials with high FOM values using Sm‐doped PMN‐PT. (a) Crystallographic structure of pristine PMN‐PT. (b) Modified crystal architecture upon samarium doping in the PMN‐PT matrix. (c) XRD patterns with corresponding Rietveld refinement results. The Sm‐doped composition exhibits an MPB, featuring a coexistence of monoclinic and tetragonal phases. (d) Polarization‐electric field (*P*–*E*), (e) strain‐electric field (*S–E*). (f) Variation in the key functional properties of Sm‐doped PMN‐PT with respect to different PT content ratios. The maximum FOM is achieved at the 32PT composition. (g) Spatial distribution of the piezoelectric coefficient within a wheel‐shaped piezoelectric structure. (h) Mapped relative permittivity across the wheel structure. (i) Localized FOM values derived from the combined piezoelectric and dielectric properties at various positions on the wheel.

Efficient piezoelectric energy harvesting relies critically on the synergy between advanced piezoelectric materials and optimized structural designs. A key determinant of the energy conversion performance is the figure of merit (FOM) of the material, which is defined as the product of the piezoelectric charge coefficient (d_33_) and the piezoelectric voltage coefficient (g_33_). As the electrical power generated during direct piezoelectric energy harvesting is proportional to the FOM, achieving a high d_33_ × g_33_ value is essential for maximizing the output. Lead‐based piezoelectric materials have significantly progressed, with the piezoelectric coefficients (d_33_) evolving from approximately 300 pC/N in the 1950s to over 1000 pC/N in recent years through refined compositional tuning and advanced crystal‐growth techniques [34, 35, 36]. Among these, PMN‐PT, a prominent soft relaxor piezoelectric material, displays an exceptional piezoelectric performance, making it particularly suitable for energy‐harvesting applications (Figure 2a). Piezoelectric materials located near the morphotropic phase boundary (MPB) typically exhibit enhanced properties owing to increased lattice asymmetry and polarization domain rotations [37, 38, 39, 40, 41]. However, studies based on MPB design strategies have continued for several decades and are now facing inherent limitations in further improving material performance. Recently, rare‐earth doping at the perovskite A‐site (Pb‐site) has further enhanced piezoelectric performance by inducing local structural heterogeneity (Figure 2b) [42, 43, 44, 45, 46, 47, 48]. Samarium doping is particularly advantageous for energy‐harvesting applications owing to its high FOM [49, 50].

To optimize the material composition for the maximum FOM, we synthesized samples with a constant Sm doping concentration (1.5 mol%) while varying the PbTiO_3_ (PT) content. X‐ray diffraction (XRD) analyses revealed the phase compositions of the synthesized samples (Figure 2c; Figure S4). Samples with 30–32 mol% PT exhibited the coexistence of monoclinic and tetragonal phases, indicating that the optimal piezoelectric performance is typically observed near the MPB. The cross‐sectional SEM image of the Sm‐doped PMN‐32PT sample (Figure S5) further confirmed dense microstructure and well‐formed grains, indicating successful sintering and high material integrity. Notably, the MPB composition of PMN‐PT (undoped) is known to occur at approximately 35 mol% PT [51]. However, in our study, the introduction of 1.5 mol% Sm dopant shifted the MPB toward a lower PT content, with the MPB behavior observed at approximately 32 mol% PT. This compositional shift reflects the influence of Sm‐induced local lattice distortion, which locally favors the formation of tetragonal regions and consequently shifts the MPB toward a lower PT content. Furthermore, the polarization‐electric field (*P–E*) hysteresis loops (Figure 2d; Figure S6), strain‐electric field (*S–E*), and current‐electric field (*I–E*) curves (Figure 2e), demonstrate the relaxor ferroelectric characteristics inherent to PMN‐PT‐based materials. The frequency dependence of the dielectric maximum temperature (T_m_) was evaluated to assess the relaxor behavior of the Sm‐doped PMN‐32PT (Figure S7). The T_m_ exhibited clear frequency dispersion, with T_m, 100 Hz_ = 124°C and T_m, 100 kHz_ = 131°C. This 7°C shift toward higher temperatures with increasing frequency indicates relaxor ferroelectric behavior, which is associated with the dynamics of polar nanoregions and a diffuse phase transition [52]. After poling based on the respective *P–E* hysteresis behavior and Curie temperatures (T_c_), the 32PT composition demonstrated the highest FOM among all the tested samples (Figure 2f; Figure S8), which is attributed to the optimized phase coexistence facilitating maximum domain mobility.

To practically apply the optimized Sm‐doped PMN‐PT material to our DC‐PG, we fabricated a wheel‐shaped structure as shown in Figure S9. Given that material uniformity directly influences the stability and quality of the generated DC output, a detailed characterization was conducted at intervals of 10° around the wheel, assessing the piezoelectric coefficients (Figure 2g), permittivity (Figure 2h), and FOM (Figure 2i). In addition, considering the structural distinctions between conventional pellet‐type samples and ring‐shaped devices, the dielectric constants were evaluated in detail (see Note S2). Despite its larger scale relative to conventional pellets, our wheel‐shaped piezoelectric generator exhibited excellent performance, including a piezoelectric coefficient (d_33_) of 860 ± 9 pC/N, a relative permittivity of 4814 ± 176, and an FOM of 17.37 ± 0.75 × 10^−^^1^ ^2^ m^2^/N. Importantly, the combination of high performance with minimal variation underscores outstanding uniformity and successful scalability, validating the practical suitability of the material for DC energy‐harvesting applications.

### 2.3 DC Power Generation Characteristics

The performance of a DC‐PG fundamentally depends on its material characteristics, mechanical design, and operational conditions (Note S1). To experimentally evaluate its performance, we fabricated a wheel‐shaped piezoelectric ceramic generator integrated with bearings. The performance was characterized using a custom‐built testing stage (Figure S10 and Video S1). The generator maintained stable contact with an aluminum (A6061 alloy) bottom plate, a material frequently employed in precision optical applications. To ensure reliable electrical contact, a metallic electrode was positioned between the wheel and the aluminum plate as the bottom electrode.

The performance characterization began with a comparative assessment between our DC‐PG and a similarly sized commercial electromagnetic induction generator under identical operational conditions (Figure S11). Although electromagnetic induction generators are widely used, their effectiveness is significantly limited by the inherent mechanical resistance owing to Lenz's law, which causes substantial performance and efficiency losses at low rotational speed. Specifically, a higher power output in electromagnetic generators necessitates either increasing the coil windings or enhancing the permanent magnet strength, inevitably resulting in greater mechanical resistance and a strong trade‐off relationship between performance and torque. The commercial electromagnetic generator used in this study required a relatively low driving torque of approximately 0.2 N·m.

**FIGURE 3:**
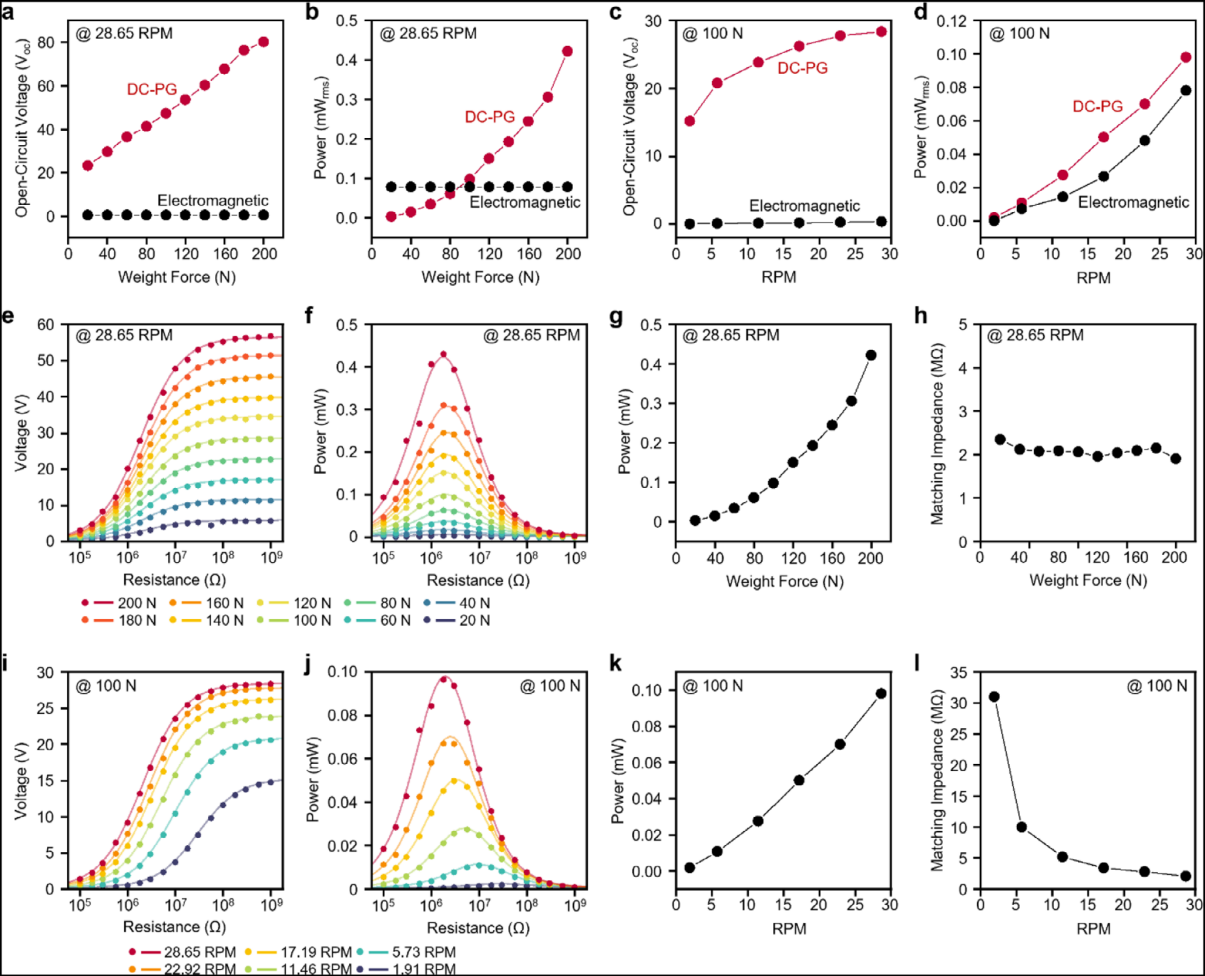
Comparative performance analysis and parameter optimization of the wheel‐shaped DC‐PG. (a–d) Benchmarking against a commercially available electromagnetic induction generator of comparable size and operational conditions. The DC‐PG exhibits a marked increase in (a) open‐circuit voltage and (b) output power with applied axial force, and maintains superior (c) voltage and (d) power across varying rotational frequency, highlighting its efficiency advantages over conventional systems that suffer substantial losses from voltage boosting. (e–h) Performance characterization of the DC‐PG under varying axial forces at optimized load conditions. The (e) output voltage and (f) power profiles across external load resistances identify optimal matching, with the (g) maximum output power exhibiting a quadratic dependence on applied force, consistent with theoretical predictions. (h) The internal resistance variations provide further insights into force‐dependent impedance behavior. (i–l) Rotational frequency‐dependent performance evaluation. The (i) voltage and (j) power outputs across different load resistances reveal optimal operational conditions, whereas (k) the peak power increases linearly with rotational frequency. (l) The internal resistance decreases with higher rotational frequency, aligning well with theoretical expectations and informing future device optimization.

Under controlled conditions, Figure 3a,b shows the measurements at a constant rotational frequency of 28.65 RPM while the axial weight force was varied. As theoretically predicted by this relationship (V ∝ ε_r_·p·T), our DC‐PG exhibited a linear increase in the open‐circuit voltage and output power with increasing applied force, distinctly contrasting the constant of the electromagnetic generator independent of axial force.

Subsequently, Figure 3c,d shows the data collected under a fixed axial force of 100 N while varying the rotational frequency. Both generators exhibited an increased output at higher rotational frequency; however, the voltage of the electromagnetic generator remained below 1 V, which is significantly lower than that of our DC‐PG. Low‐voltage outputs from electromagnetic generators pose practical challenges and require voltage boosting (typically 3–5 V) for practical applications. Such boosting necessitates DC‐DC converters, typically achieving a conversion efficiency of less than 10% at low power levels, further reducing the usable power. Conversely, our DC‐PG naturally generates sufficient voltage, enabling highly efficient (>95%) step‐down DC‐DC conversion, thus markedly enhancing practical energy availability and widening the performance gap between the two technologies. Under identical operating conditions, the open‐circuit voltage of our DC‐PG was approximately 85 times higher, and the output power was 1.25 times greater than that of the electromagnetic generator, demonstrating its superior energy‐harvesting capability.

Further evaluation involved impedance matching under varying axial forces (Figure 3e–h) and rotational frequency (Figure 3i–l). At a constant rotational frequency of 28.65 RPM (Figure 3e–h), the output power demonstrated a quadratic relationship with the applied force, consistent with the theoretical expectations, as both the voltage and current increased with the axial force. At a constant axial force of 100 N, variations in rotational frequency (Figure 3i–l) revealed a linear increase in the output power, which is consistent with the theory that rotational frequency predominantly influences current generation. Notably, the internal resistance decreased at higher rotational frequency, as theoretically anticipated (Figure 3l). Collectively, these comprehensive experimental results validate our theoretical model (Note S1) and highlight the significant performance and reliability advantages of our wheel‐shaped DC‐PG, which is particularly suitable for compact, low‐torque, and energy‐efficient applications.

### 2.4 Single Electrode Performance for Real‐World Applications

Piezoelectric generators traditionally employ a dual‐electrode configuration, with electrodes positioned on opposite faces of the material to facilitate the charge flow induced by polarization changes under mechanical stress. In wheel‐shaped generators, the conductive bottom surface typically serves as an external electrode, enabling effective DC generation. However, this approach is impractical for everyday applications where most environmental surfaces are insulating. To address this limitation, we implemented a novel single‐electrode configuration that utilize only an internal electrode integrated within a wheel‐shaped piezoelectric element.

**FIGURE 4:**
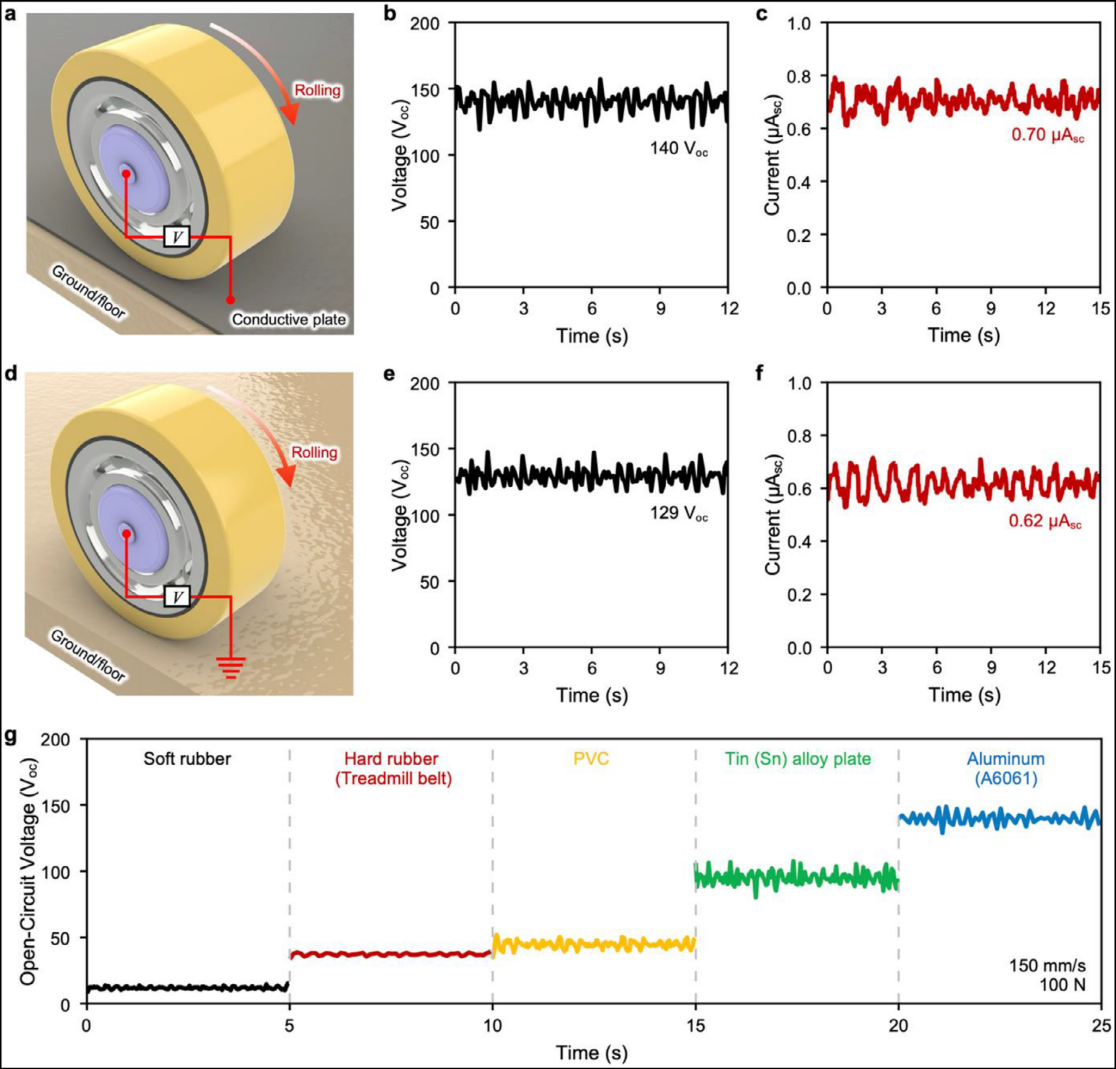
Comparison between conventional double‐electrode and single‐electrode DC‐PG. (a) Experimental setup for measuring a conventional piezoelectric generator employing a plate with conductive metal deposited on both surfaces. (b) Open‐circuit voltage and (c) short‐circuit current measured from the double‐electrode configuration. (d) For enhanced practicality, an alternative approach using a plate with no surface deposition was investigated. Notably, despite the floor being electrically insulating, the system generated substantial electrical output. (e) Open‐circuit voltage and (f) short‐circuit current results confirm that the single‐electrode configuration can achieve a meaningful performance without significant energy loss. (g) Performance comparison of the DC‐PG using plates with varying Young's moduli. Materials with lower Young's moduli (i.e., softer materials) generally produced lower open‐circuit voltages, whereas stiffer materials with higher Young's moduli yielded higher voltage outputs, demonstrating the importance of mechanical stiffness in optimizing generator performance.

Figure 4a–c illustrates the traditional double‐electrode setup, wherein a conductive aluminum (A6061) plate serves as the external electrode. Under these conditions, the measured open‐circuit voltage and short‐circuit current reached approximately 140 V and 0.7 µA, respectively. In contrast, our innovative single‐electrode configuration (Figure 4d) relies solely on the internal electrode and utilizes induced charge via intrinsic polarization fluctuations within the piezoelectric material [53, 54, 55]. Despite the initial expectations of reduced efficiency, experimental results obtained on insulating substrates (Figure 4e,f) demonstrated a robust performance, achieving an open‐circuit voltage of 129 V (92% relative to the dual‐electrode setup) and a short‐circuit current of 0.62 µA (88% relative to the dual‐electrode conditions). These outcomes confirm the practicality and effectiveness of our single‐electrode approach, enabling stable DC generation even in the absence of conductive surfaces, thereby significantly expanding potential applications. Note S3 provides additional details on the underlying operating principles of single‐electrode generation.

Furthermore, recognizing that real‐world flooring materials possess diverse mechanical properties, we systematically evaluated their influence on the generator performance (Note S4). Mechanical interactions between the wheel‐shaped piezoelectric generator and flooring materials directly depend on each the Young's modulus of each material, which significantly affects the output performance (Table S1) [56, 57, 58]. Softer materials, such as rubber, experience substantial deformation, increasing the contact area but absorbing mechanical stress, thus substantially reducing the effective pressure transfer to the piezoelectric element. Conversely, rigid materials with a high Young's modulus exhibit minimal deformation and thus smaller contact areas, enhancing the pressure transmission and significantly boosting the voltage output (Figure 4g). These findings illustrate the crucial role played by flooring stiffness in optimizing the DC‐PG performance. Overall, the demonstrated success of the single‐electrode configuration marks a notable advancement toward practical applications, offering enhanced adaptability and consistent performance across various real‐world scenarios. To evaluate the durability of the piezoelectric DC‐PG, a cyclic lifespan test was performed for 10 000 rolling cycles under identical operating conditions (Figure S12). After 10 000 cycles, the output power showed a decrease of approximately ∼11% compared to the initial value. This relatively small degradation indicates that the rolling‐induced compressive loading introduces minimal mechanical fatigue, confirming the robustness of the proposed DC‐PG design. Figure S13 demonstrates that the single‐electrode DC‐PG maintains stable output on both dry and wet ground conditions, showing only a minor voltage reduction with V_OC_ values of approximately 128 V for the dry condition and 122 V for the wet condition, indicating only a minor reduction under waterish environments. These results confirm that the single‐electrode DC‐PG does not rely on a conductive ground as a counter‐electrode and maintains robust DC generation even under varying surface conditions.

### 2.5 Demonstration of Powering an IoT Sensor Using a Wheel DC‐PG

Accurate, real‐time tracking remains a persistent challenge in mobility and logistics systems, particularly in shared transportation services such as public bicycles and baggage handling within airports. In shared‐bike services, localization errors frequently cause rental and return validation issues. Luggage loss remains a common problem in airports. On average, approximately 7.6 bags are mishandled per 1000 passengers. Given the annual global air passenger count of 3.8 billion, this represents a massive logistical and economic burden. These issues highlight the growing demand for compact, maintenance‐free, and self‐powered tracking technologies that can be seamlessly embedded within existing infrastructure, without adding bulk or requiring frequent recharging. More importantly, such systems should operate without additional human effort or external energy input, emphasizing their self‐generation and lack of additional torque.

**FIGURE 5:**
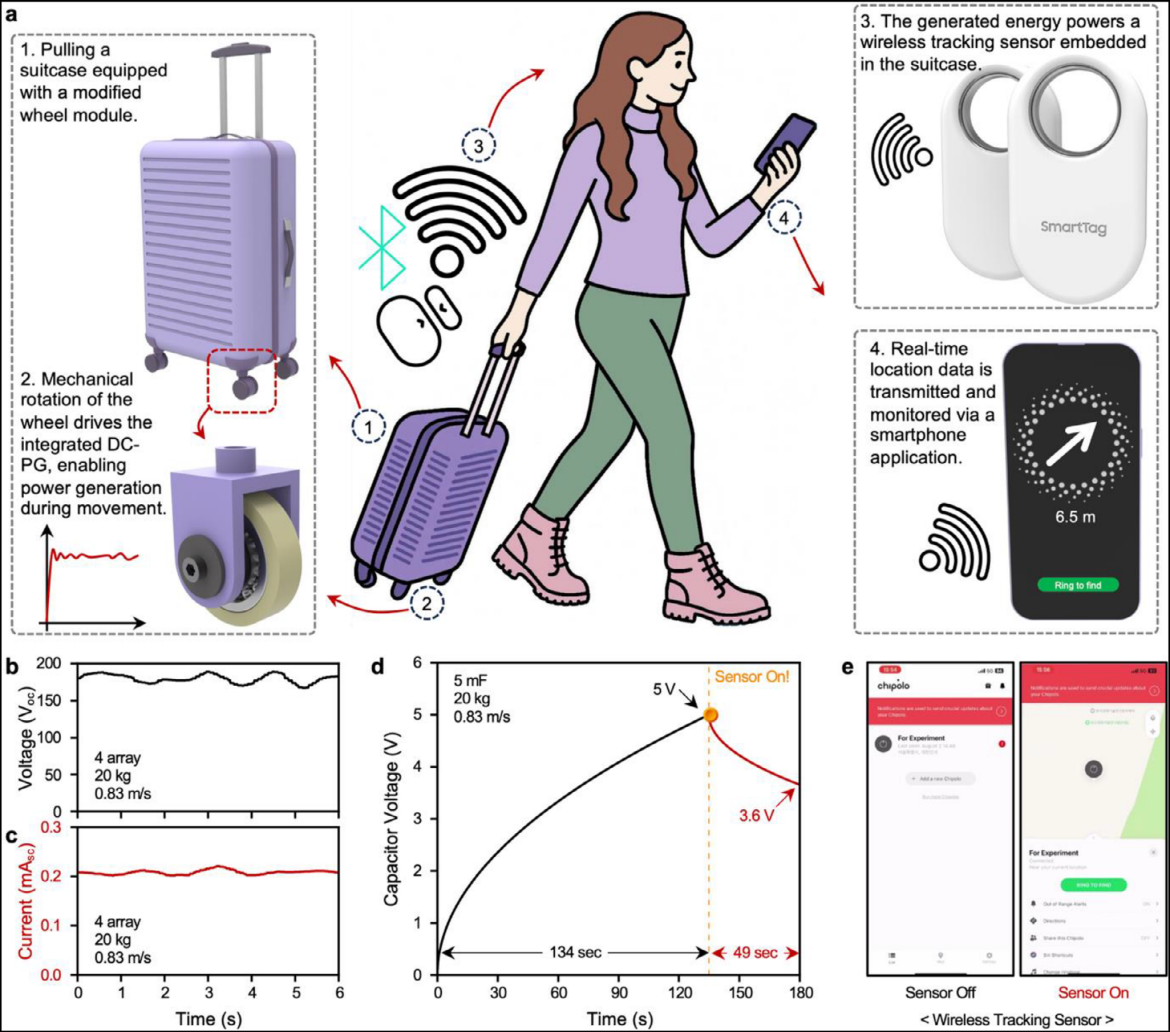
Demonstration of a self‐powered smart luggage system enabled by in‐wheel DC‐PGs. (a) Conceptual illustration of a smart suitcase application powered by in‐wheel DC‐PG, which drive a wireless location‐tracking sensor that communicates directly with a smartphone in real time. (b) Measured open‐circuit voltage, and (c) short‐circuit current when all four wheels of the suitcase are replaced with DC‐PGs and operated on the treadmill. (d) Voltage profile of a 5 mF during sensor charging. The capacitor reached 5 V from 0 V in just 134 s, enabling a continuous 49 s operation of the sensor. (e) Real‐time demonstration of successful sensor‐to‐smartphone communication powered solely by the energy harvested through the DC‐PGs.

Figure 5a illustrates the overall concept of a fully self‐powered smart luggage system without additional torque. In this configuration, the original suitcase wheels are replaced with custom‐designed wheel‐shaped DC‐PGs, that harvest electricity solely from rolling motion. This energy directly powers a Bluetooth‐enabled location‐tracking sensor embedded within a suitcase, enabling real‐time communication with a smartphone without the need for an external power source or battery.

To demonstrate the practical utility of our DC‐PG in real‐world scenarios, we developed a fully integrated wireless real‐time location tracking system embedded in a suitcase (Figure S14). A 3D‐printed mounting structure was used instead of standard wheels to install the DC‐PGs (Figure S15), allowing each wheel to generate DC electricity independently during motion. The outputs from all four wheels were connected in parallel, with each channel incorporating diodes to prevent reverse current flow and ensure stable and continuous energy harvesting. A buck‐type DC‐DC converter was used to stabilize the combined output to 3.7 V (Figure S16), thereby creating a reliable power supply suitable for low‐power IoT electronics. Notably, DC‐PGs inherently produce a DC with consistent polarity regardless of the rolling direction, eliminating the need for rectification circuitry in AC‐based generators.

To evaluate the system performance under realistic conditions, we conducted experiments using a standard 20 kg suitcase moving at 3–5 km/h on a treadmill, mimicking typical airport usage. In this scenario, the integrated DC‐PG system produced an open‐circuit voltage of ∼175 V and a short‐circuit current of ∼0.21 mA. The harvested energy was used to charge a 5 mF capacitor, which reached 5 V within 134 s of movement. After the suitcase was stopped, the stored energy was used to power the tracking sensor until the voltage dropped to 3.6 V, confirming a stable and sustained operation (Figure 5b–d; Video S2). These results highlight the robustness of the generator under a continuous dynamic load and demonstrate its capacity to power real‐world electronics based solely on mechanical motion. The peak charging power of the system was measured to be ∼4.28 mW corresponding to an energy density of 0.61 mW/cm^3^ (Figure S17), whereas the combined power consumption of the sensor and converter was calculated to be ∼0.123 mW based on the capacitor discharge profiles. This wide power margin confirmed the suitability of the generator for continuous sensor operation under typical travel conditions.

To further emphasize the benefits of the direct DC output, we compared our wheel‐shaped DC‐PG with a conventional pellet‐shaped piezoelectric generator of equal volume (3 cm diameter, 1 cm thickness). When both devices were subjected to equivalent cyclic loading conditions (20 kg at 5.3 Hz), the DC‐PG charged a 5 mF capacitor to 4.83 V in 120 s, whereas the AC generator, which requires rectification, only reached 3.53 V (Figure S18). This illustrates the superior energy utilization and reduced loss inherent to DC‐PG.

This demonstration not only validates the technical feasibility of our DC‐PG system but also reveals its potential as a compact, maintenance‐free, and battery‐less power solution for next‐generation IoT applications. Its seamless integration into existing mobility platforms has paved the way for real‐world deployment in logistics, shared mobility services, and smart city infrastructures, where autonomous and self‐sustaining devices are becoming increasingly critical components.

## 3 Conclusion

In this paper, we introduced an approach for piezoelectric energy harvesting by developing a wheel‐shaped piezoelectric generator capable of directly producing high‐quality DC output from mechanical rotation. Departing from conventional AC‐generating systems based on vibration or bending, our design leveraged continuous rotational motion to achieve inherently unidirectional current generation with extremely low torque, which represents an innovation that redefines the architecture of piezoelectric generators. This advancement was made possible by the development of a specialized piezoelectric material, Sm‐doped PMN‐PT ceramic optimized for radial poling within a circular geometry. This material exhibited a high FOM and superior electromechanical performance, enabling a robust and stable DC output under dynamic conditions. Beyond material and structural innovations, we conducted a series of rigorous experiments under realistic operational scenarios to assess the performance of the generator in practical applications. These include variable load conditions, rotational speeds, and dual‐ and single‐electrode configurations. Notably, the single‐electrode setup demonstrated an output comparable to that of conventional designs while facilitating implementation on electrically insulating surfaces. This significantly expands the scope of the real‐world applications. Most importantly, we validated the practical value of the generator through a fully integrated battery‐free application: a smart suitcase system featuring in‐wheel DC‐PGs powering a real‐time wireless location‐tracking sensor. The system harvested energy directly from the motion of the suitcase to charge a capacitor and continuously operated a commercial sensor module, eliminating the need for external power sources or AC‐DC conversion. The generator was seamlessly embedded in standard wheel dimensions without additional size, weight, or structural modifications. This demonstrated the readiness of the system for integration into daily mobile platforms. More broadly, this study overturned the long‐standing assumption that piezoelectric generators must produce AC by introducing a new class of DC‐PGs tailored for real‐world motion. Through the synergy of advanced materials, innovative device architectures, and successful system‐level integration, this technology has emerged as a promising, sustainable, compact, and maintenance‐free energy solution for future IoT and mobility applications.

## 4 Experimental Section

### 4.1 Wheel DC‐PG Synthesis of Samarium‐Doped Lead Magnesium Niobate‐Lead Titanate (Sm‐Doped PMN‐PT)

Pb_0.9775_Sm_0.015_[(Mg_1/3_Nb_2/3_)_(1‐x)_Ti_x_]O_3_ (x = 0.26–0.34) was synthesized using the two‐step columbite precursor method. The initial step involved the synthesis of magnesium niobium oxide (MgNb_2_O_6_), followed by the synthesis of lead oxide (PbO), titanium oxide (TiO_2_), and samarium oxide (Sm_2_O_3_) in accordance with their respective molar ratios, using a precision balance (Analytical Balance MS204S, Mettler Toledo, USA) for accurate measurements.

#### 4.1.1 MgNb_2_O_6_ Synthesis

Dried MgO powder (342793, Sigma–Aldrich, USA) was subjected to heat treatment at 1000°C for 2 h in a furnace (SiC Box Furnace, Yokogawa, Japan) to effectively eliminate any adsorbed moisture from the powder. The dried MgO powder was combined with Nb_2_O_5_ (NBO06PB, Kojundo Chemical Laboratory Co., Ltd., Japan) and mechanically milled for 24 h. The milling process was conducted at 110 rpm using a ball mill machine (HBM‐5003RC, Hantech Co., Ltd., Korea), employing a polypropylene jar and zirconia ball (yttrium stabilized zirconia oxide balls, Samhwa, Korea). Ethanol was used as the processing medium. The resultant mixed powder was subjected to a drying process at 100°C for 24 h in a forced convection drying oven (SH‐FDO149, Samheung, Korea). To ensure the complete removal of ethanol, an Alumina (Al_2_O_3_) crucible with a capacity of 300 cc (Samhwa, Korea) was employed during this drying stage. The dried powder was subjected to calcination at 1100°C for 4 h. After the calcination, a second round of mechanical milling was performed at 110 rpm 24 h.

#### 4.1.2 PVA Binder

A Poly(vinyl alcohol) (PVA) binder was employed to enhance the cohesion among the powder particles, facilitating the fabrication of ceramics with the desired shape. A mixture of 95 mL of distilled (Di) water and 5 g PVA (348406, Sigma–Aldrich, USA) was prepared. The prepared PVA solution was subjected to heat and continuous stirring at 95°C for 24 h using a digital hotplate stirrer (MSH‐20D, Daihan Scientific Co., Ltd., Korea) equipped with a magnetic bar, operating at 180 rpm. The solution was cooled to room temperature.

#### 4.1.3 Sm‐Doped PMN‐PT Synthesis

A mixture of MgNb_2_O_6_ powder, PbO (PBO09PB, Kojundo Chemical Laboratory Co., Ltd), TiO_2_ (248576, Sigma–Aldrich, USA), and Sm_2_O_3_ (120658, Sigma–Aldrich, USA) was prepared by accurately weighing the components using an analytical balance (Model MS204S, Mettler Toledo, USA). The precursor mixture was mechanically milled at 110 rpm for 24 h using a ball mill. The milled powder was calcinated at 850°C for 2 h within a furnace. Following the calcination, a second mechanical milling step was performed at 110 rpm for 24 h. A small quantity of PVA was mixed with the milled material and dispersed using a standard test sieve (200 µm, CG‐20341‐75, Chung Gye, Korea). The resulting mixture was pressed into a disk shape using a circular mold with a diameter of 12 mm and a hydraulic laboratory press (HLP‐C12, Hantech Co., Ltd, Korea) under a pressure of 10 MPa. The binder was subsequently removed by heat treatment at 600°C for 2 h within a furnace. The samples were sintered at 1275°C for 2 h in a furnace using an alumina crucible. The polishing was conducted to a thickness of 0.7 mm and a diameter of approximately 10 mm in accordance with IEEE standards using sandpaper (CC261, Misumi, Japan) to measure the material properties.

#### 4.1.4 Sm‐doped PMN‐PT Ring Manufacturing

The Sm‐doped PMN‐PT powder was pressed using a custom‐made cylindrical mold with an outer diameter of 50 mm, an inner diameter of 40 mm, and a thickness of 10 mm using a manual hydraulic press (Model 3925, Carver Inc., USA) under a pressure of 6 t. To eliminate the binder, the compacted samples were subjected to a heat treatment at 600°C for 2 h within a furnace. The samples were further processed by subjecting them to cold isostatic pressing at a pressure of 200 MPa for 10 min using the cold isostatic pressing method (Model ECIP‐120‐360, Energyn Inc., Korea). Finally, the processed samples were sintered in a furnace at 1100°C for 24. Following sintering, the samples were coated with a silver paste (S‐311J, IMD, Korea). Subsequently, they were subjected to firing at 600°C for 10 min and polarized through corona poling at 3.5 kV/mm in silicon oil at 120°C for 30 min. All the samples were characterized after a minimum of 24 h had elapsed since the poling process.

### 4.2 Characterization of Material Properties

A ferroelectric tester (Precision Multiferroic Tester, Radiant Technologies, Inc, USA) was used to measure the polarization‐electric field (*P–E*) hysteresis curve. For this measurement, the Sm‐doped PMN‐PT ceramic pellets were connected to a wired socket and dipped in silicone oil to prevent the break‐down of the thrown air. The dielectric constant was measured using a precision LCR meter (4284A, HP, USA) from 100 Hz to 1 MHz with a physical property measurement system (PPMS‐9T, Quantum Design, USA) from room temperature to 300°C. The piezoelectric coefficient (*d_33_*) was measured using a quasi‐static d33‐m (d_33_/d_31_ meter, ZJ‐6BJ, Beijing Jingke Technology Development). XRD (D8 Advance, Bruker, USA) with Cu K_α_ radiation was used to analyze the crystal structure. The microstructure was analyzed using scanning electron microscopy (SEM, Inspect F50, FEI, USA).

### 4.3 Electrical Performance Characterization

The performance of the wheel‐type DC‐PG was characterized using a custom mechanical system in which a step motor was used to move linearly periodically. The electrical output was measured using a four‐channel digital phosphor oscilloscope (DPO4014B, Tektronix) with a voltage probe (P2220 Voltage probe, Tektronix) with an input impedance of 10 MΩ, an electrometer/high‐resistance meter (6517B, Keithley), and a low‐noise current pre‐amplifier (SR570, Stanford Research Systems). The LabVIEW program was used to control the custom mechanical system and electrometer/high‐resistance meter.

### 4.4 3D Printing

Complex shapes were designed using the SolidWorks software, assembly models were created, and various design parameters, including material selection, were adjusted. The model was then transformed into a printable format for 3D printing using a 3D slicer software (IdeaMaker, RAISE3D, USA). During slicing, the printer settings, layer thickness, printing speed, and placement of the support structures were adjusted. Finally, the sliced STL file was sent to a 3D printer (Single Plus‐320C, Cubicon) to create a physical model.

### 4.5 Construction of Real‐Time Wireless Location Tracking System

A circuit constructed with a 5 mF capacitor and an energy generator breakout module (LTD3588, Linear Technologies) was used to supply the rated voltage (3.3 V_dc_) to the sensor network. A real‐time location tracking system (Chipolo ONE Bluetooth Key Finder, Chipolo d.o.o.) was used to track the location in real time, and tracking information was obtained in real time using a mobile phone application provided by the manufacturer. For the long‐term driving experiment at a human walking speed (for an adult male, 1.5 m/s), a driving experiment was conducted using a treadmill (HERA‐9000A, Health‐One Comp.). For application experiments using travel carriers, commercially available carriers (suitcase, Wheelpack Comp.) were used, and the wheel‐fixing parts were specially manufactured using a 3D printer (Single Plus‐320C, Cubicon). The weight of the travel carrier was tested by loading weights inside it based on the upper limit of the actual aircraft baggage weight (approximately 23 kg or less in the case of Korean Air).

## Funding

This work was supported by the National Research Foundation of Korea (NRF) grant funded by the Korea government (MSIT) (RS‐2024‐00448865 and RS‐2025‐25429261). This research was partially supported by Korea University (K2521191)

## Conflicts of Interest

The authors declare no conflicts of interest.

## Supporting information




**Supporting File 1**: advs73941‐sup‐0001‐SuppMat.docx.

**Supporting File 2**: advs73941‐sup‐0002‐VideoS1.mp4.

**Supporting File 3**: advs73941‐sup‐0003‐VideoS2.mp4.

## Data Availability

The data that support the findings of this study are available from the corresponding author upon reasonable request.
