# Supplementary material for: In‐Wheel Piezoelectric DC Power Generator With Zero Resistive Torque
Source: Adv Sci (Weinh). 2026 Jan 28;13(18):e22932. doi: 10.1002/advs.202522932 (PMC13042964; doi:10.1002/advs.202522932)
Supplement: Supplementary file 1 — Supporting File 1: advs73941‐sup‐0001‐SuppMat.docx. [file ADVS-13-e22932-s001.docx]

Supporting Information

In-wheel Piezoelectric DC Power Generator with Zero Resistive Torque

Hyun Soo Kim^†^, Hyunseok Song^†^, In Woo Oh^†^, Dong-Gyu Lee, Tae Kyoung Yoon, Jeyeon Lee, Byoung Jin Yoon, So-Min Song, Iman M. Imani, Seohyun Cho, Seong Jin Kim, Chong-Yun Kang, Sahn Nahm, Yong Seok Park, Kyung-Hoon Cho, Jungho Ryu, Jeong Min Baik, Jun Chen, Sunghoon Hur* and Hyun-Cheol Song*

**1. Supplementary Notes**

**Note S1. Theoretical Model of a Wheel-Shaped Direct-Current Piezoelectric Generator**

The working mechanism of DC-PG can be fundamentally understood based on the charge-generation behavior of conventional piezoelectric generators. When a mechanical force is applied to a piezoelectric material, it induces an electric charge on the electrodes, as described by the basic constitutive relationship.

| $Q=d_{33}\cdot F=d_{33}\cdot p\cdot A$ | (S1) |
| --- | --- |

where *Q* is the charge, *F* is the force, *p* is the pressure, and *A* is the area. The introduction of a capacitor model allows the relationship between charge, voltage, and capacitance to be represented as follows:

| $Q=C\cdot V=d_{33}\cdot p\cdot A$ | (S2) |
| --- | --- |

where *C* is the capacitance, and *V* is the voltage. The key difference between conventional piezoelectric generators (C-PGs) and the proposed DC-PG is the interpretation of mechanical input. In the C-PG, the electrical output is driven by temporal variations in the applied pressure.


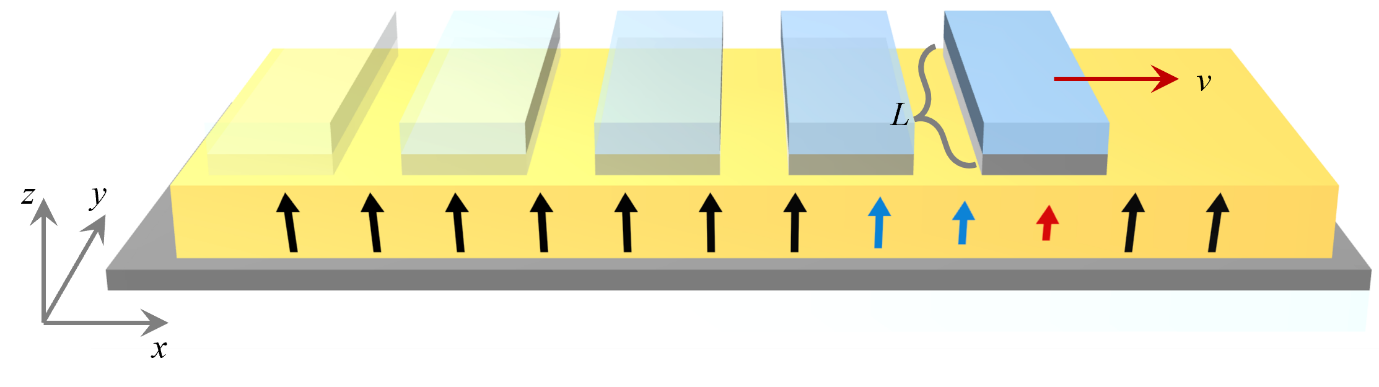


**Figure N1. Schematic of the operating principle of the DC-PG.**

In contrast, the DC-PG operates under constant pressure and generates current through the spatial modulation of the active area. This behavior can be quantitatively described by differentiating the governing equations with respect to time, as follows:

| $dQ=d_{33}\cdot d\left( p\cdot A \right)=d_{33}\cdot p\cdot dA$ | (S3) |
| --- | --- |
| $I_{sc}=\frac{dQ}{dt}=d_{33}\cdot p\cdot\frac{\partial A}{\partial t}=d_{33}\cdot p\cdot L\cdot v$ | (S4) |
| $V\cdot\frac{\partial C}{\partial t}=d_{33}\cdot p\cdot\frac{\partial A}{\partial t}$ | (S5) |
| $V_{oc}=d_{33}\cdot p\cdot\frac{\partial A}{\partial t}\cdot\frac{\partial t}{\partial C}=d_{33}\cdot p\cdot\frac{\partial A}{\partial C}=\frac{d_{33}}{\varepsilon_{33}^{T}}\cdot p\cdot T$ | (S6) |

where *L* is the length, *T* is the thickness of the piezoelectric material, $\varepsilon_{33}^{T}$ is the dielectric constant, and *v* is moving velocity. Therefore, the open-circuit voltage and short-circuit current of the DC-PG are governed by intrinsic material parameters (*d_33_*, $\varepsilon_{33}^{T}$), geometric factors (*L*, *T*), and operational velocity (*v*). A distinct feature of the DC-PG is its internal resistance behavior. Under the impedance-matched condition, where maximum power transfer occurs, the internal resistance can be analytically expressed as follows.

| $R_{in}=\frac{\left( \frac{1}{2}V_{oc} \right)}{\left( \frac{1}{2}I_{sc} \right)}=\frac{V_{\mathrm{oc}}}{I_{sc}}=\frac{T}{\varepsilon_{33}^{T}\cdot L\cdot v}$ | (S7) |
| --- | --- |

The internal resistance of the DC-PG is uniquely dependent not only on the material and structural parameters, but also on the operating velocity. Specifically, the resistance decreases inversely with increasing velocity. Based on the derived expressions, the theoretical power output can be calculated as

| $P=\frac{1}{2}V_{oc}\cdot\frac{1}{2}I_{sc}=\frac{1}{4}\cdot\frac{d_{33}^{2}}{\varepsilon_{33}^{T}}\cdot p^{2}\cdot L\cdot v\cdot T$ | (S8) |
| --- | --- |

In summary, this theoretical analysis clarifies the core operating principle of the wheel-shaped DC-PG and reveals how its electrical performance is governed by the intrinsic material parameters, structural dimensions, and motion dynamics. The derived expressions not only align with experimental observations but also provide valuable guidelines for future design and optimization. These results support the experimental results and device demonstrations presented in the main manuscript.

**Note S2. Theoretical Model for Measuring the Dielectric Constant of Ring-Shaped Piezoelectric Devices**

Typically, the permittivity of a material is determined in alignment with the IEEE standards by depositing electrodes onto both surfaces of a pellet-shaped sample with a uniform thickness. However, the piezoelectric ring examined in this study requires electrode deposition both internally and externally on the ring, followed by polarization in the radial direction, rendering conventional methods unsuitable for calculation. An intuitive approach involves leveraging the average area of the inner and outer electrode surfaces, combined with the difference between the two radii as the effective thickness for the computation. However, this strategy is only viable for significantly large rings. Therefore, to obtain a more refined permittivity measurement, we conceptualized a subsequent approach.


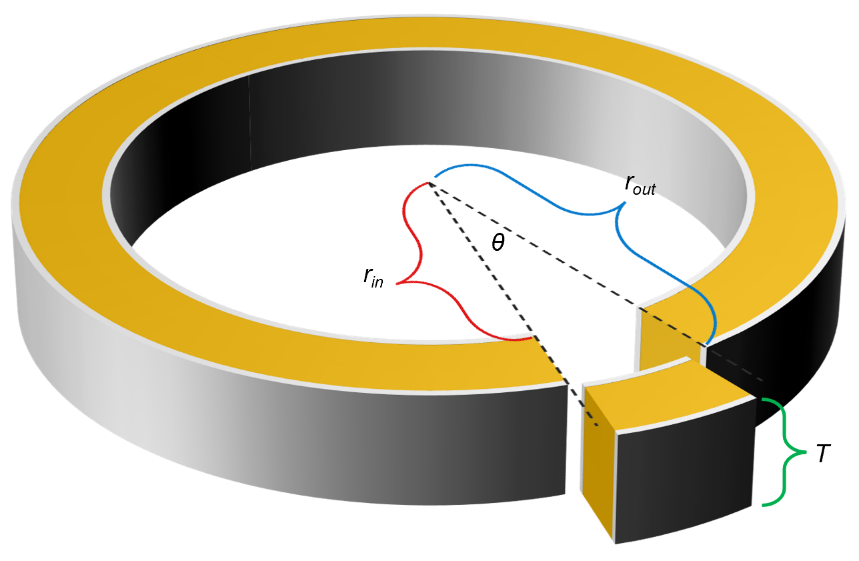


**Figure N2. Schematic of permittivity measurement for a wheel-shaped piezoelectric element.** Electrodes are deposited on the inner and outer surfaces of the ring. A Gaussian surface is constructed between them to calculate the permittivity using Gauss’s law.

Fundamentally, the most precise method involves assuming charges *Q* and -*Q* on each electrode and applying Gauss’s law from Maxwell’s equations. Accordingly, one can conceptualize a Gaussian closed surface with a radius r emanating from the center of the ring and thickness *T*, as depicted. The electric field can then be computed as follows using assumed Gaussian surface:

| $\nabla\cdot E=\frac{\rho}{\varepsilon_{0}}$ (Gauss’s law in free space) | (S9) |
| --- | --- |
| $\nabla\cdot D=\rho$ (Gauss’s law in a material medium) | (S10) |
| $\oint D\cdot dA=2\pi r\cdot T\cdot E=\frac{Q}{\varepsilon_{33}^{T}}$ | (S11) |
| $D=\frac{Q}{\varepsilon_{33}^{T}}\cdot\frac{1}{2\pi r\cdot T}$ | (S12) |

where $D$ is the electric displacement field, $\rho$ is the charge density, $\varepsilon_{0}$ is the permittivity of vacuum and air, $\varepsilon_{33}^{T}$ is the dielectric constant, $r$ is the radius of the Gaussian closed surface, and $T$ is the thickness of the piezoelectric ring. The voltage can be determined by integrating eq. (4) radially from *r_in_* to *r_out_*:

| $\left\vert V \right\vert=\int_{r_{in}}^{r_{out}} D\cdot dr = \frac{Q}{2\pi\cdot T\cdot\varepsilon_{33}^{T}}\cdot\int_{r_{in}}^{r_{out}} \frac{1}{r}\cdot dr = \frac{Q}{2\pi\cdot T\cdot\varepsilon_{33}^{T}}\cdot ln\left( \frac{r_{out}}{r_{in}} \right)$ | (S13) |
| --- | --- |

The relationship between the electrical capacitance and the voltage across the electrode terminals can be expressed as $Q=C\cdot V$. Given this association, the electrical capacitance can be derived. Furthermore, considering that the electrodes have been partitioned at a consistent angle *θ*, the resultant capacitance, factoring in this segmentation, can be formulated as follows:

| $C=\frac{Q}{V} = 2\pi\cdot T\cdot\varepsilon_{33}^{T}\cdot{ln}^{-1} \left( \frac{r_{out}}{r_{in}} \right)$ | (S14) |
| --- | --- |
| $C_{\theta} = \frac{\theta}{2\pi}\cdot C = \theta\cdot T\cdot\varepsilon_{33}^{T}\cdot{ln}^{-1} \left( \frac{r_{out}}{r_{in}} \right)$ | (S15) |

Consequently, the position-dependent permittivity of the piezoelectric ring, segmented at an angle *θ*, can be expressed as follows:

| $\frac{\varepsilon_{33}^{T}}{\varepsilon_{0}} = \frac{C_{\theta}}{\varepsilon_{0}\cdot\theta\cdot T}\cdot{ln}^{-1} \left( \frac{r_{out}}{r_{in}} \right)$ | (S16) |
| --- | --- |

using a theoretical approach, we could achieve measurements that were approximately 16% more precise than the approximation method considering the average area of the two electrodes. As the size of the ring increased, the associated error decreased, whereas a smaller ring size likely amplified the error.

**Note S3. Theoretical Analysis Method for the Operating Principles of the Single-Electrode Piezoelectric Effect Utilizing Electrical Potential Variation**

The fundamental principle behind electric generation through the piezoelectric effect can be theoretically explained by changes in the internal polarization of the piezoelectric material, leading to potential shifts at the electrodes. Polarization alterations in a material under pressure can be classified based on its elemental composition and crystalline structure.


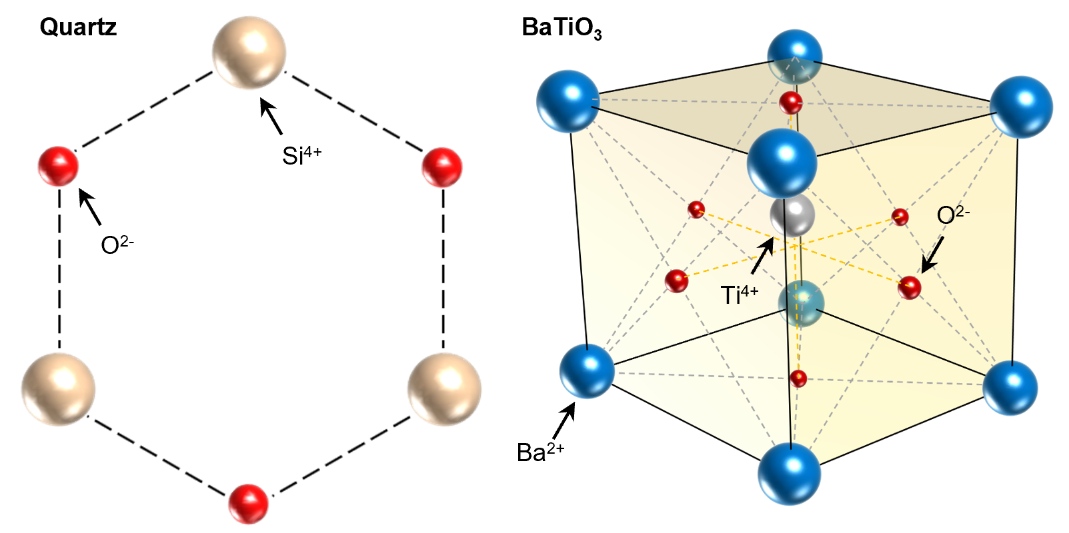


**Figure N3. Crystal structures of various piezoelectric materials.**

The quartz has a trigonal structure of SiO_2_. Owing to its electronegativity, oxygen exhibits weak negative polarity, whereas silicon exhibits weak positive polarity^[1]^. As the charge distribution centers of these two polarities coincide, no inherent polarization is observed. However, external pressure causes a distortion in its structure, leading to the separation of these centers, subsequent induction polarization. The perovskite structure is characterized by atoms positioned at the corners (A-site), body center (B-site), and face center (X-site) of an octahedron, typically with oxygen located at the X-site. Hence, it is referred to as the ABX3 structure. Notably, as the temperature of the BaTiO3 sample decreases, to minimize its structural energy, its crystalline structure transitions through rhombohedral, orthorhombic, tetragonal, and ultimately, cubic phase^[2,3]^. In the cubic phase, the atom at the B-site is situated at the center of the structure, resulting in no inherent polarization. However, reducing the temperature induces structural distortion and a shift in the position of the B-site atoms, disrupting the electrical balance and producing polarization. An exerting external force induces changes in the crystal structure, subsequently modifying its polarization.

This polarization produces an electric field that increase the energy of the overall system^[4]^. Hence, if free charges are present, they would move to screen the electric field generated by polarization, reducing the energy of the system and enhancing its stability. This phenomenon typically occurs on the surfaces or boundaries of materials with existing polarization, often by depositing metal on both ends of the ferroelectric material to provide free charges at the interface. When stress is applied to a piezoelectric material, the magnitude of its polarization changes, subsequently altering the electric field. This modification in the charge required for screening prompts surplus charges to move through a connected circuit, which is the principle upon which a piezoelectric generator operates.


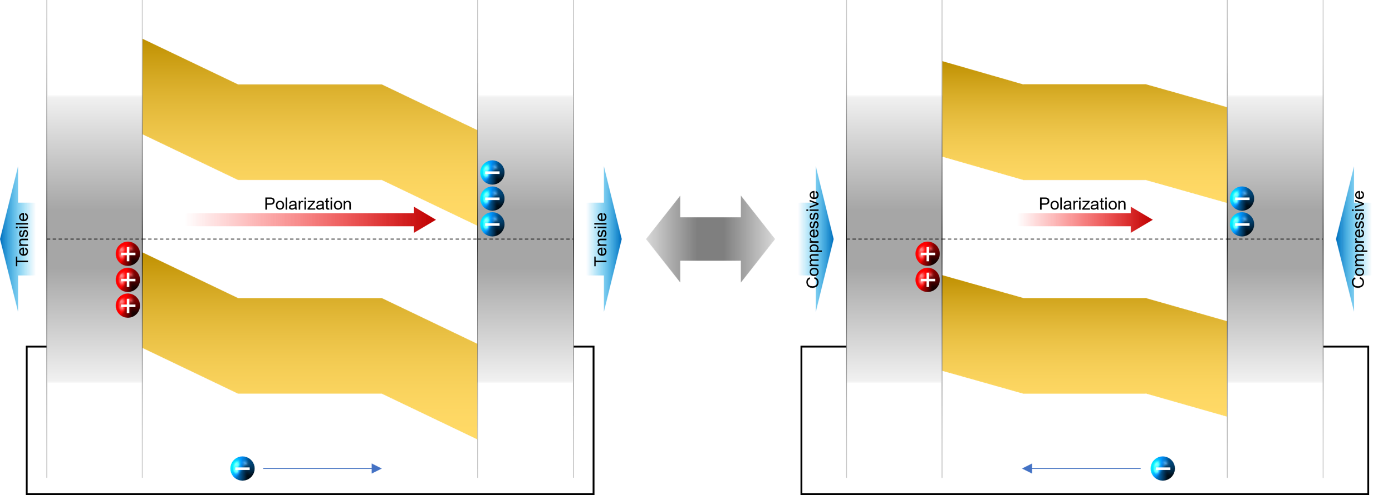


**Figure N4. Band diagram illustrating the piezoelectric effect in a conventional double-electrode configuration.**


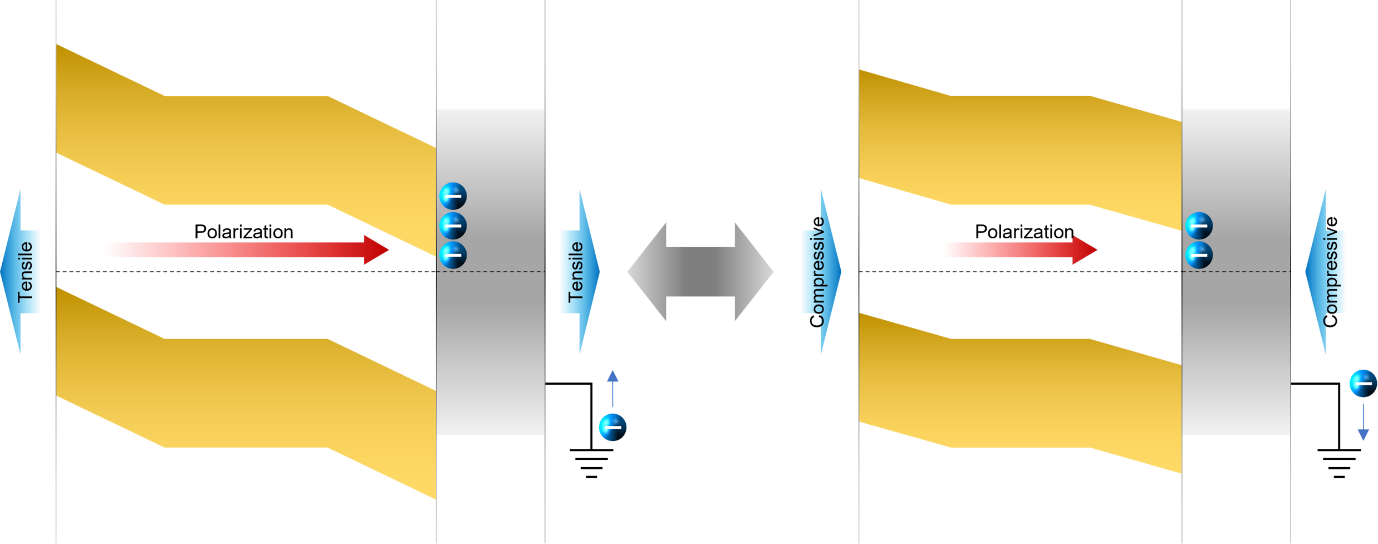


**Figure N5. Band diagram of the piezoelectric effect under a single-electrode configuration.**

Considering the operational principle of the piezoelectric material, it is discernible that implementing a piezoelectric generator does not necessarily require electrodes to be deposited on either side; it could function feasibly with an electrode deposited only on one side. A single-electrode piezoelectric generator was constructed by depositing an electrode on one surface, whereas the opposite side was connected to ground. In this single-electrode scheme, charge movement is prompted by potential changes on the electrode surface of the piezoelectric material in compared with the static electrical potential of the ground. This approach is particularly advantageous in scenarios where it may be challenging to stack electrodes at both ends, such as miniaturized generators. In addition, it has proven beneficial in hybrid systems combined with other generators, such as triboelectric generators, or in piezoelectric sensors designed to detect gaseous substances.

**Note S4. Modelling of Performance Variations Based on the Material Properties of the Contact Surface in a Wheel-Shaped Direct-Current Piezoelectric Generator**

The performance of a DC-PG is directly proportional to the voltage and current with respect to the magnitude of the pressure applied to the piezoelectric material. Consequently, one must consider not only the physical properties of the piezoelectric material but also the physical attributes of the floor. Given that the wheel-shaped DC-PG employs a piezoelectric ring, its contact area with the ground is linear. Precisely defining this contact area is challenging; however, it is conceivable to predict the contact area based on the floor material by referencing Young’s modulus.


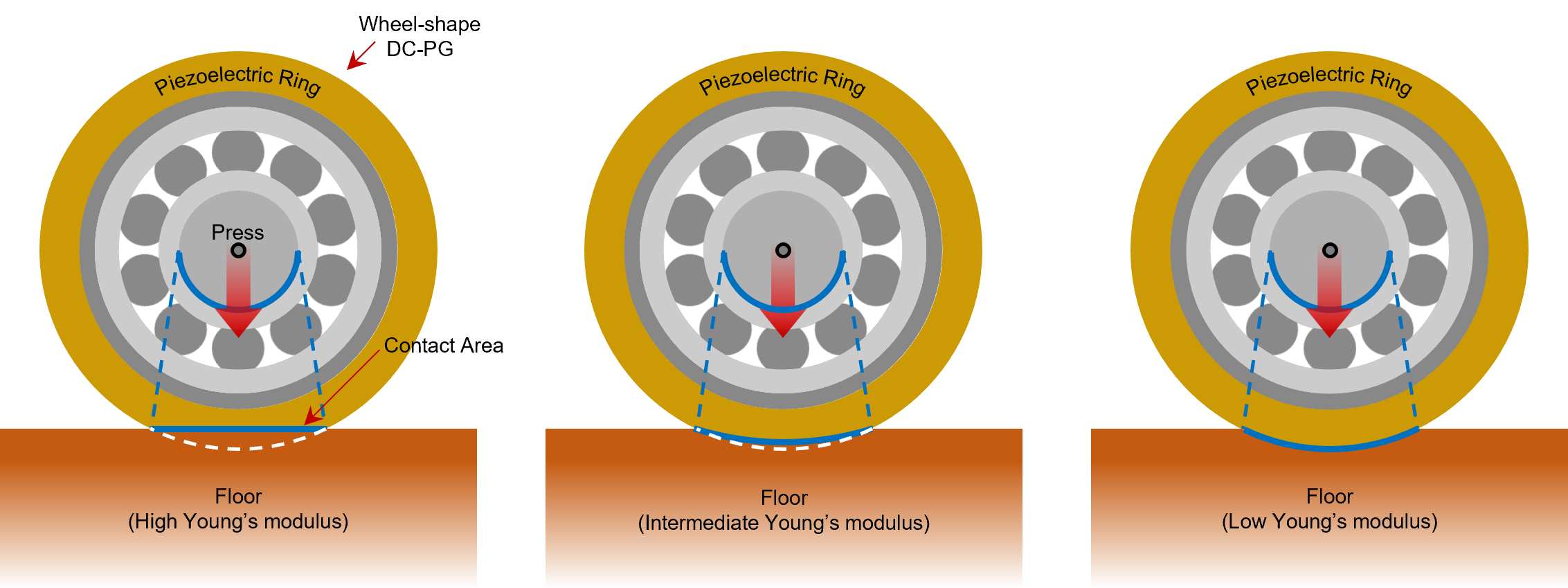


**Figure N6. Mechanical deformation of the contact surface as a function of material stiffness.**

When the Young’s modulus of the floor is exceedingly high, nearly all mechanical deformation is expected to occur within the piezoelectric ring, resulting in a flat shape that closely mirrors the floor profile, thereby minimizing the contact area. Conversely, if the Young’s modulus of the floor is extremely low, the contact area exhibits a curved shape that closely resembles the curvature of a piezoelectric ring, maximizing the contact surface. The most realistic scenario involves both the piezoelectric ring and floor with slight deformations, representing an intermediary behavior between the two extreme cases. Thus, the contact area varies with the Young’s modulus of the floor, leading to changes in the pressure exerted on the piezoelectric ring. This variation can explain the distinct tendencies of the DC-PG with different floor materials, as depicted in Figure 4g. Consistent with the predictions, the DC-PG performance diminishes when the Young’s modulus of the floor is lower, and conversely, the DC-PG performance increases as the Young’s modulus of the floor increases.

**2. Supplementary Figures**


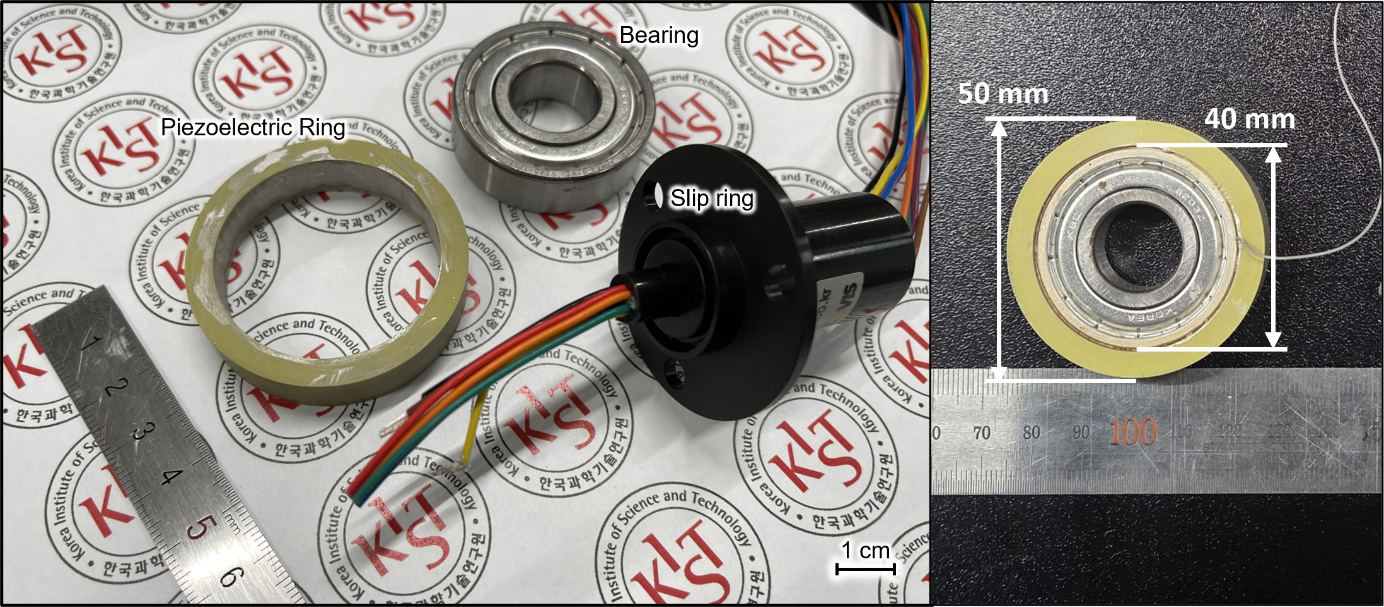


**Figure S1.** **Photograph illustrating the components of a wheel-shaped DC-PG.** It comprises a ring-shaped piezoelectric material, bearings for rotational motion, and a slip ring designed to prevent the twisting of wires. The Sm-doped PMN-PT piezoelectric ring has outer diameter of 50 mm, inner diameter of 40 mm and thickness of 10 mm.


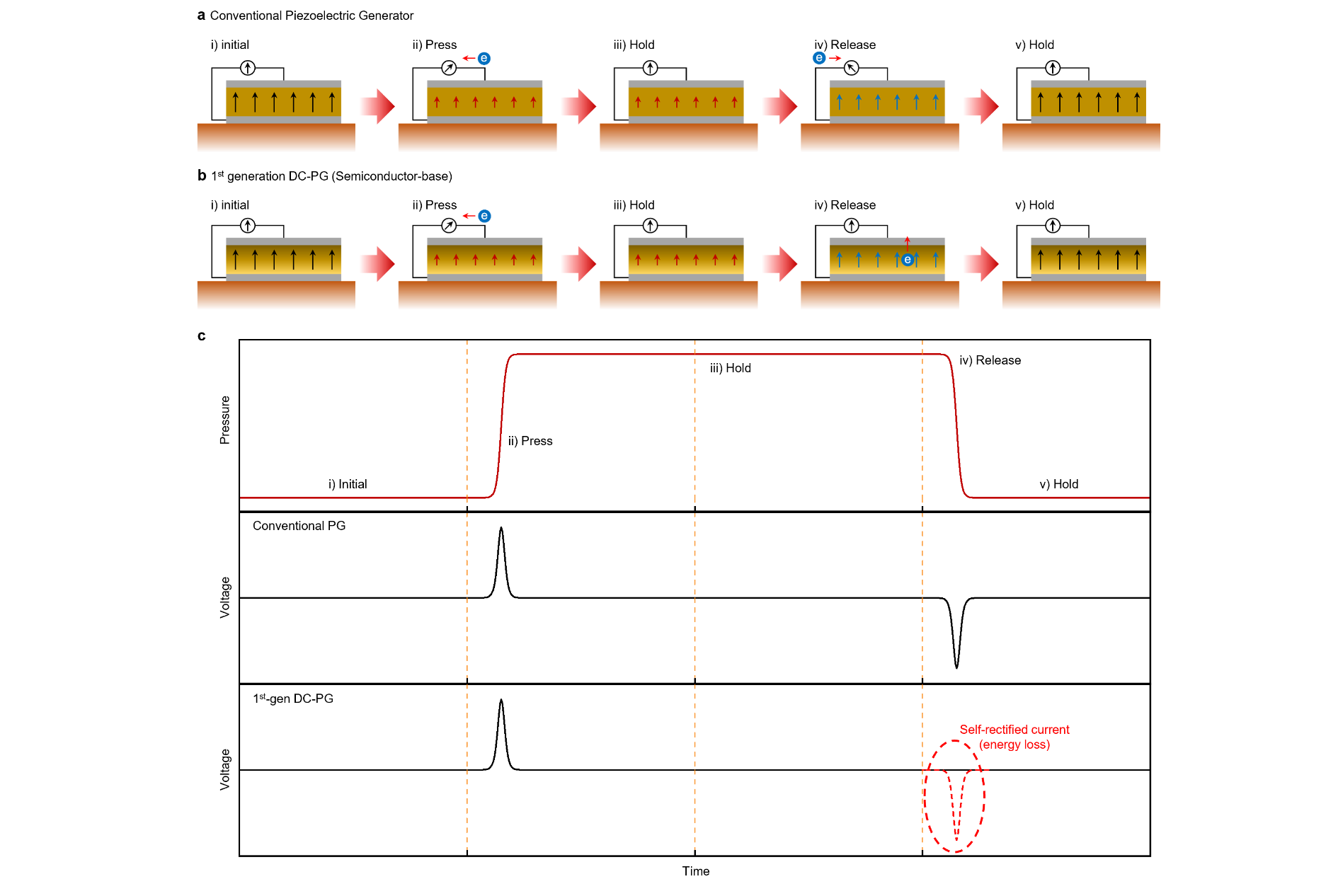


**Figure S2. Operational principles of the classical piezoelectric generator and the self-rectifying piezoelectric generator utilizing semiconductor-based piezoelectric materials, described as follows: a)** External pressure induces changes in the internal polarization, leading to the flow of electric current in the external circuit. **b)** The semiconductor-based piezoelectric generator leverages its internal p-n junction to produce leakage in one direction, thus ensuring that the current flows through the circuit in only one direction, effectuating DC. **c)** They exhibit two distinct voltage-generation tendencies in response to external pressure. As the generated voltages are of a pulse nature, they possess high peak voltages; however, their root mean square (RMS) values are notably low. Additionally, the semiconductor-based piezoelectric generator regulates reverse currents through internal leakage, leading to significant energy loss.


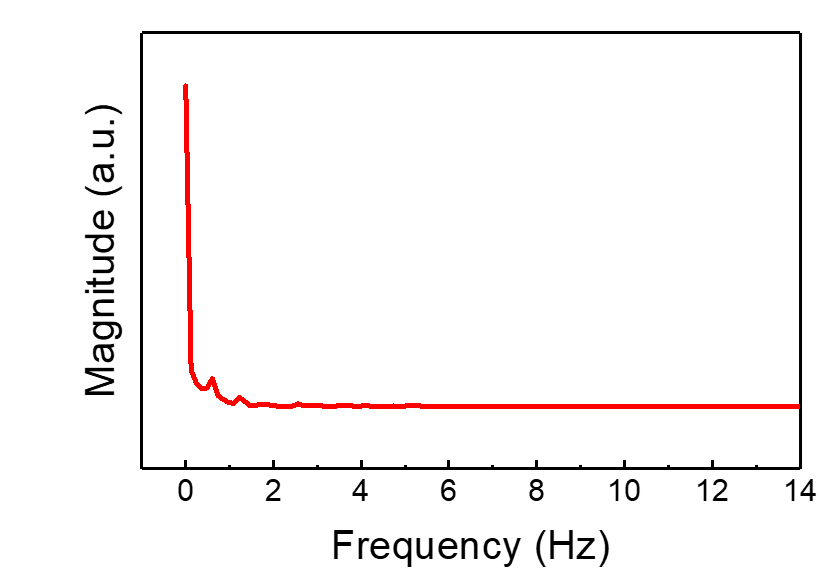


**Figure S3.** Frequency-domain analysis (Fast Fourier Transform, FFT) calculated from the open-circuit voltage output in **Figure** **1c**.


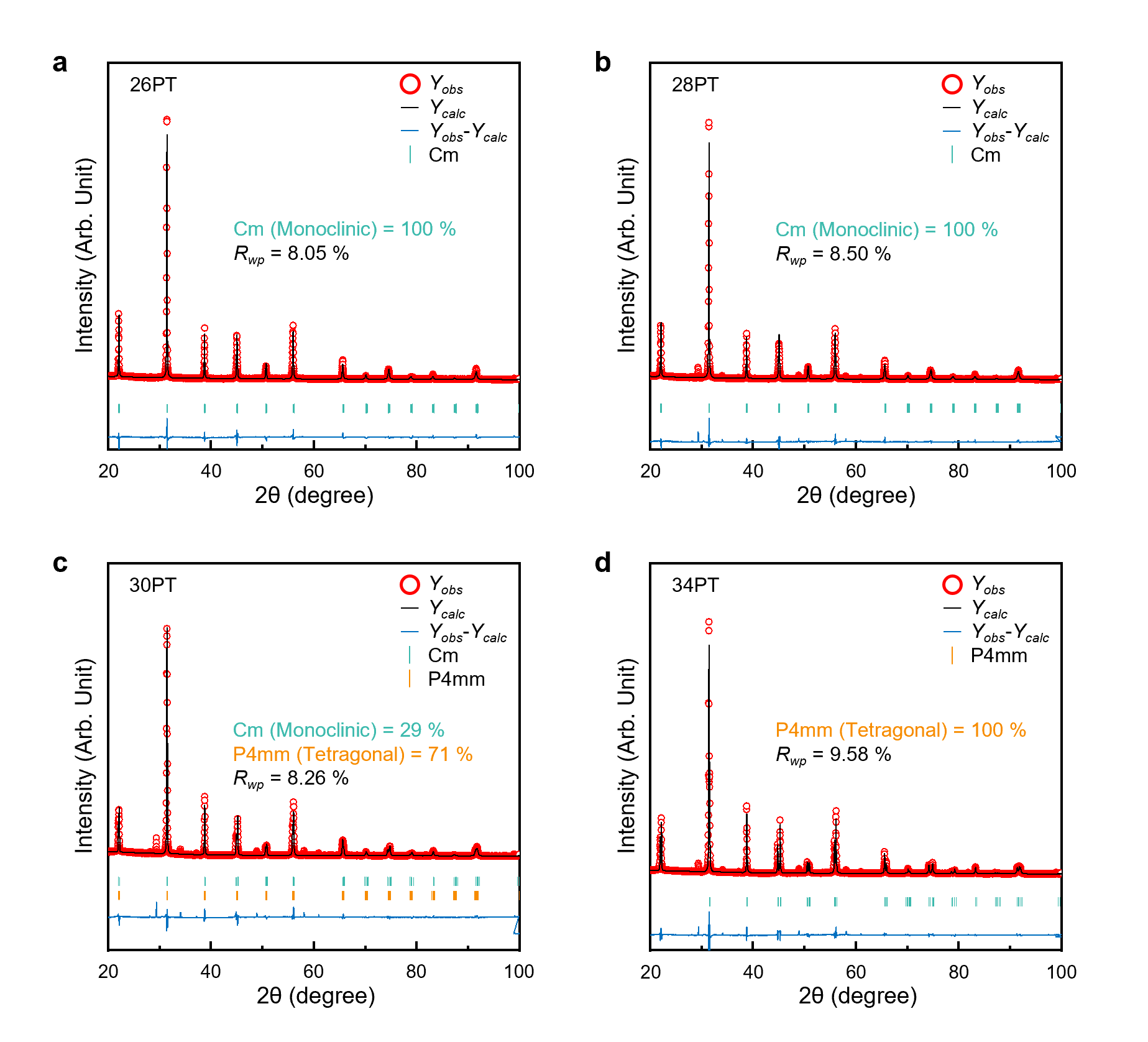


**Figure S4. XRD data and Rietveld phase analysis of Sm-doped PMN-PT with varying PT ratios. A)** 26PT and **b)** 28PT exhibit a monoclinic single phase, whereas **c)** 30PT and 32PT show an MPB composition with a mixture of monoclinic and tetragonal phases. **D)** From 34PT onward, a tetragonal single phase is observed.


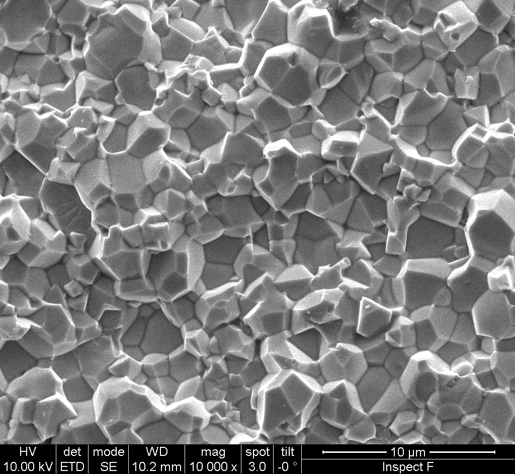


**Figure S5. Cross-sectional SEM image of Sm-doped PMN-32PT composition.**


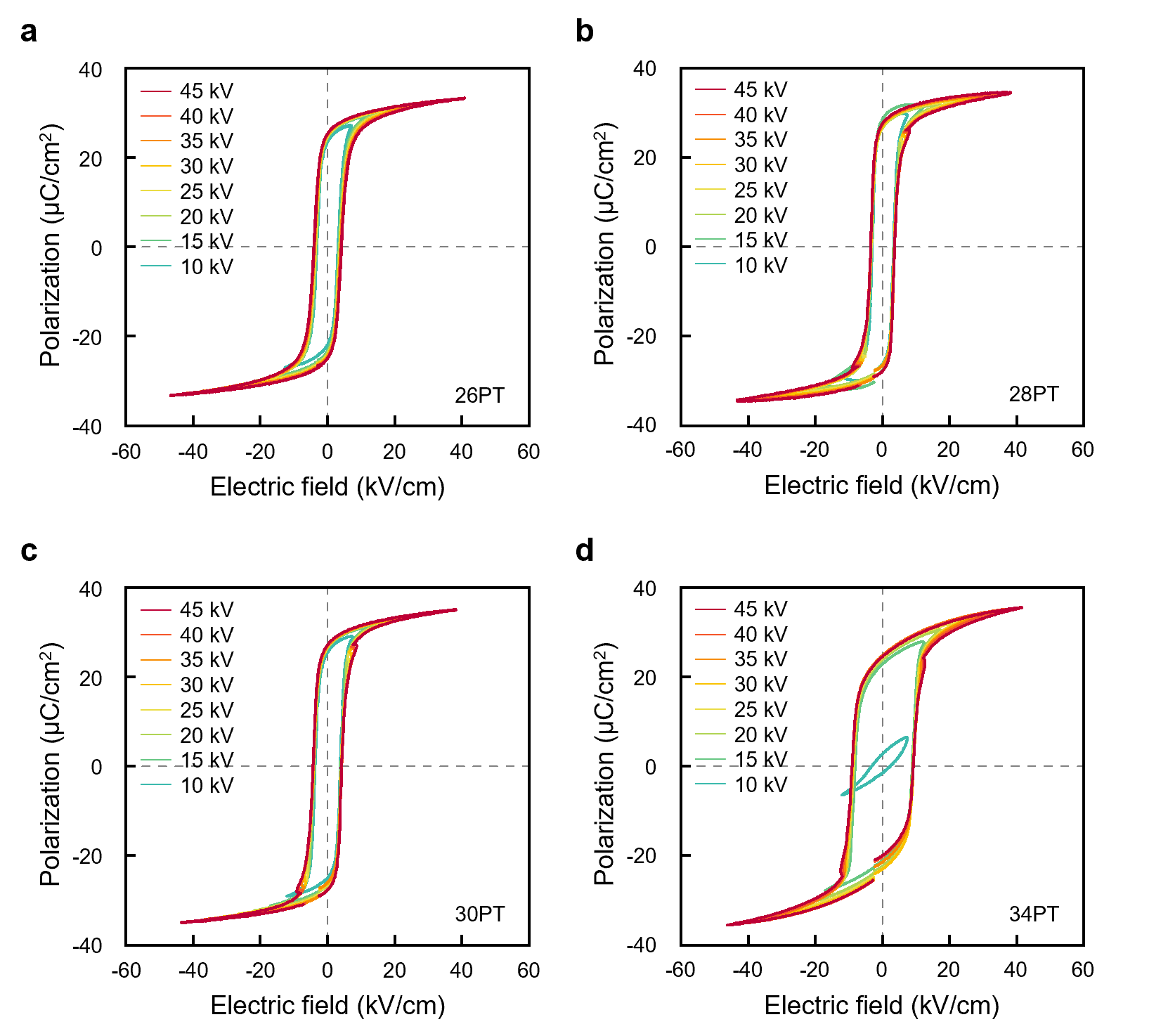


**Figure S6. Polarization–electric field hysteresis curves for Sm-doped PMN-PT as a function of PT ratio are presented. a)** 26PT, **b)** 28PT, **c)** 30PT, and **d)** 35PT ratios. As the PT ratio increases, the material properties transition slightly from hard to soft characteristics.

**
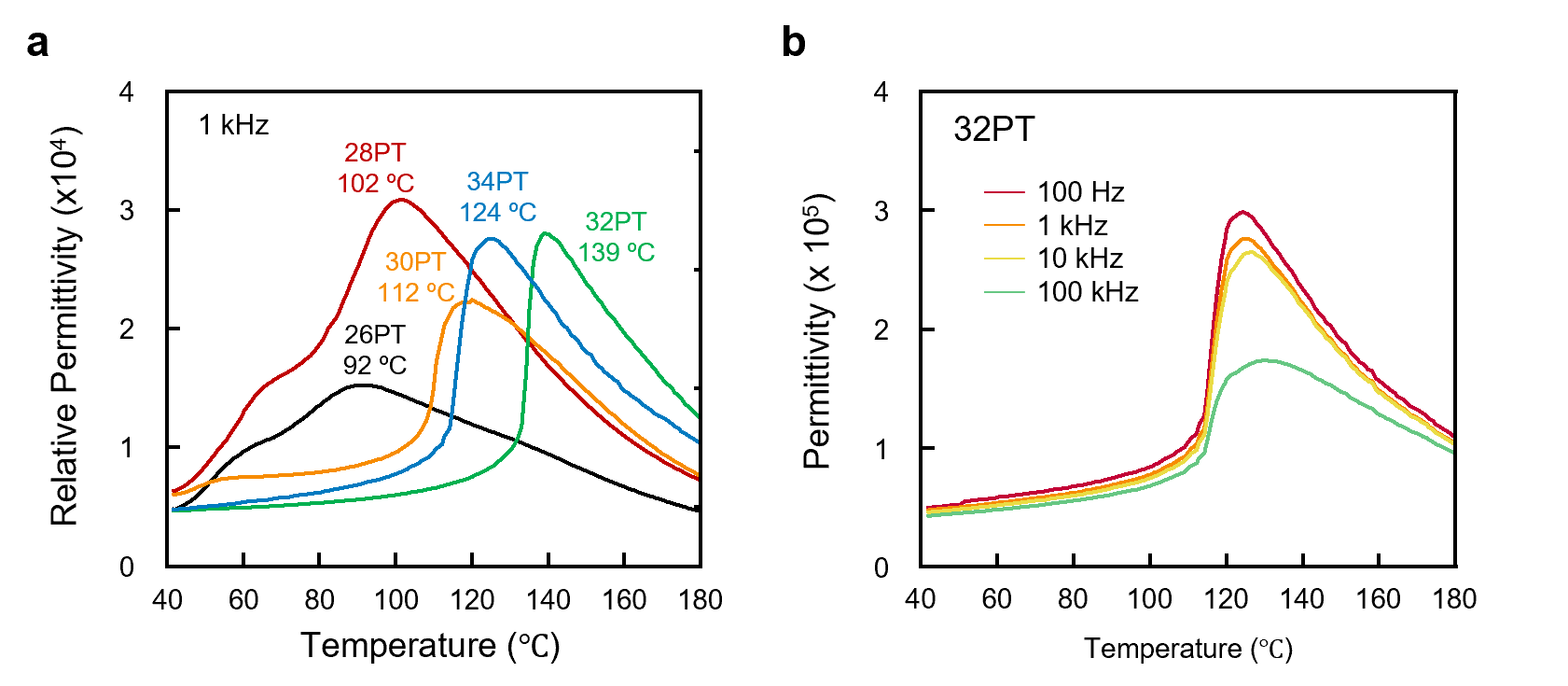
**

**Figure S7. Variation of T_c_ in Sm-doped PMN-PT. a)** As the PT ratio increases, Tc increases from 92 °C to 139 °C. **b)** The increase in T_c_ with increasing frequency confirms the relaxor composition.


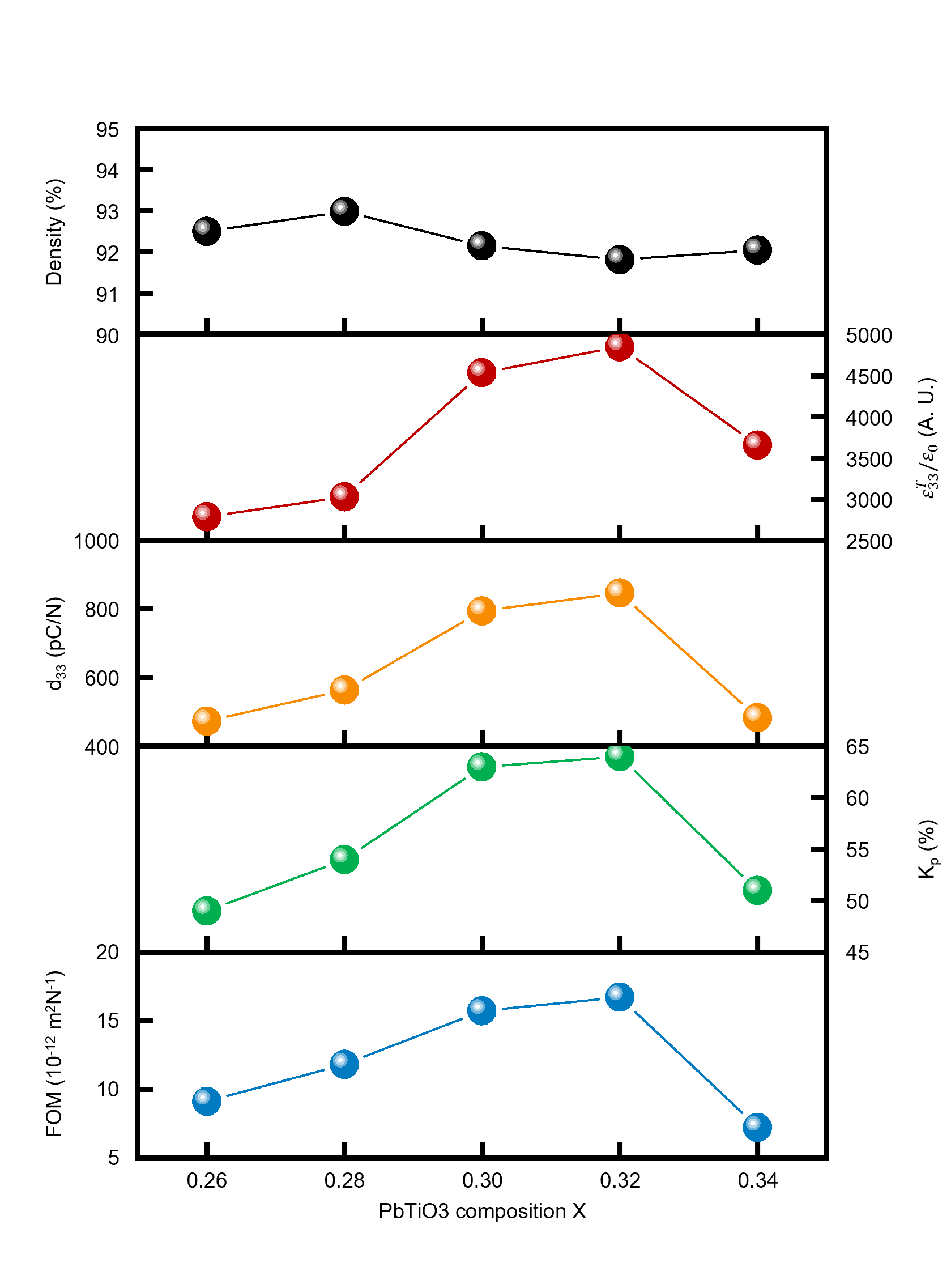


**Figure S8. Variation of properties in Sm-doped PMN-PT with different PT ratios.** A piezoelectric DC generator was fabricated using the 32PT composition, which exhibited the highest FOM.


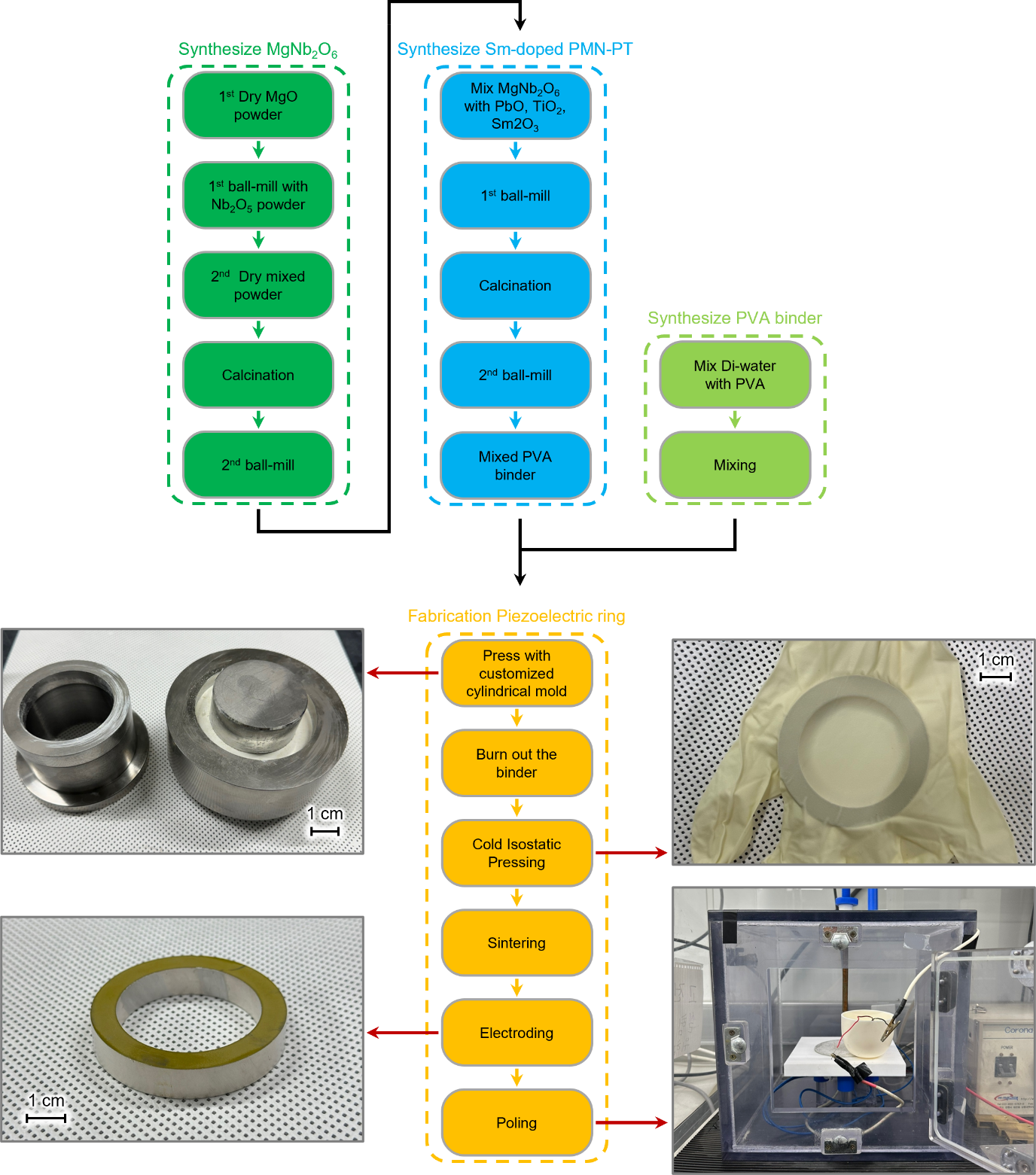


**Figure S9. Fabrication process of the piezoelectric ring using Sm-doped PMN-PT**


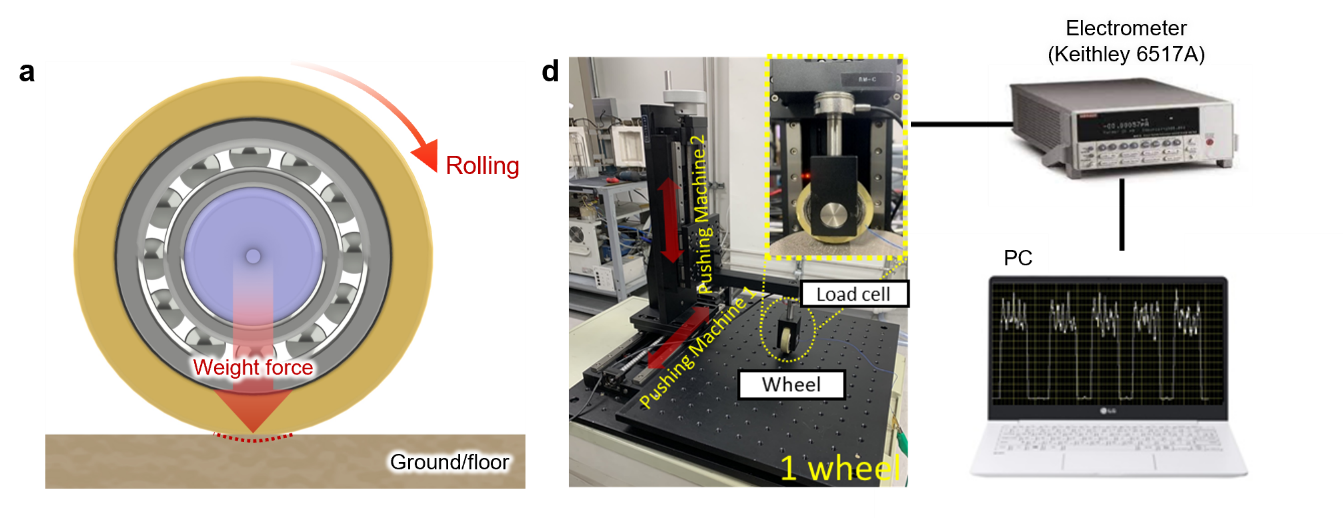


**Figure S10. Experimental setup for the performance evaluation of the DC-PG. a)** Schematic depicting the experimental configuration used for parameter-dependent measurements, including variations in applied force and travel speed. **b)** Photograph of the actual measurement setup used for device characterization.


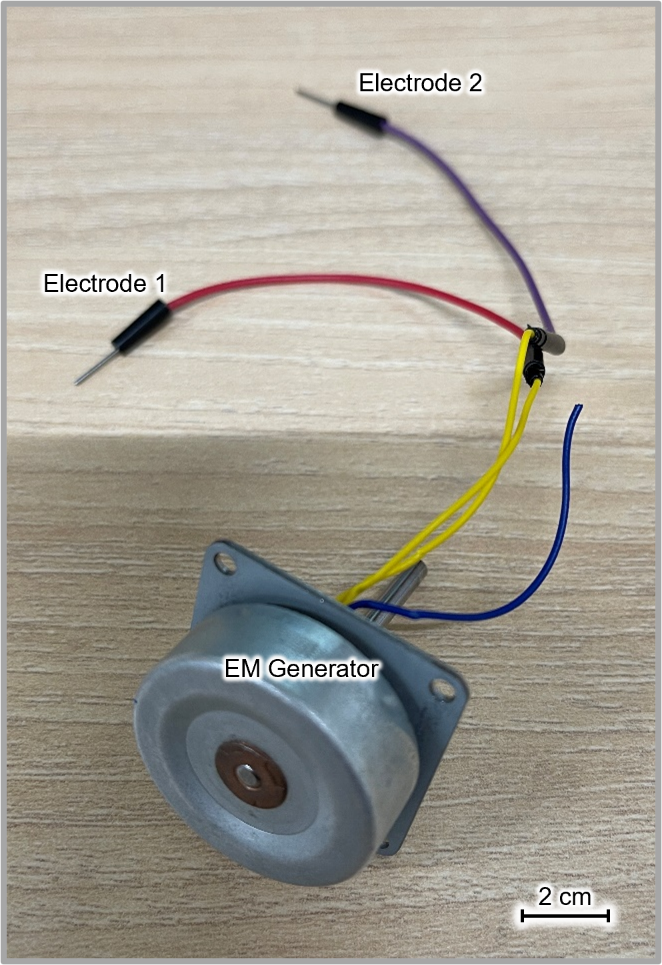


**Figure S11. Wheel-shaped DC-PG alongside a commercially available electromagnetic conduction generator, which was utilized for a performance comparison.**


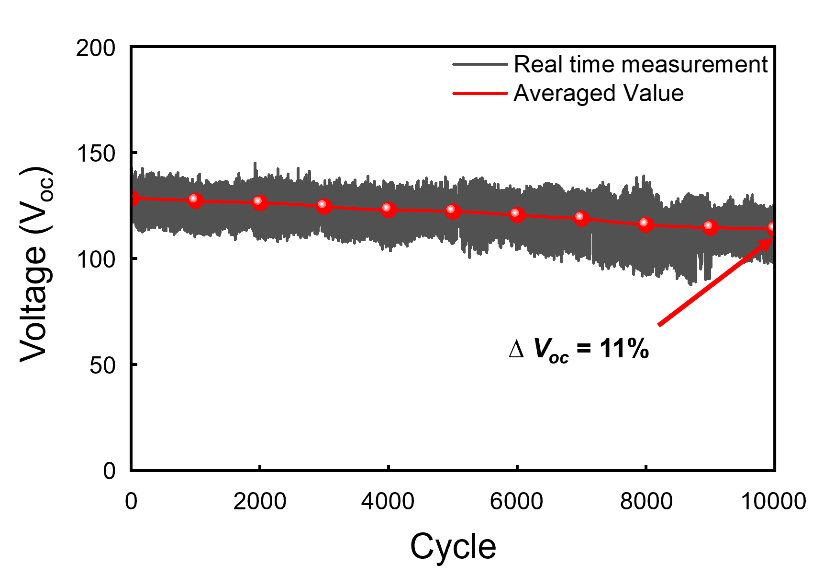


**Figure S12. Fatigue performance with 10,000 continuous rolling cycles** **of the in-wheel direct-current piezoelectric generator (DC-PG).**

**
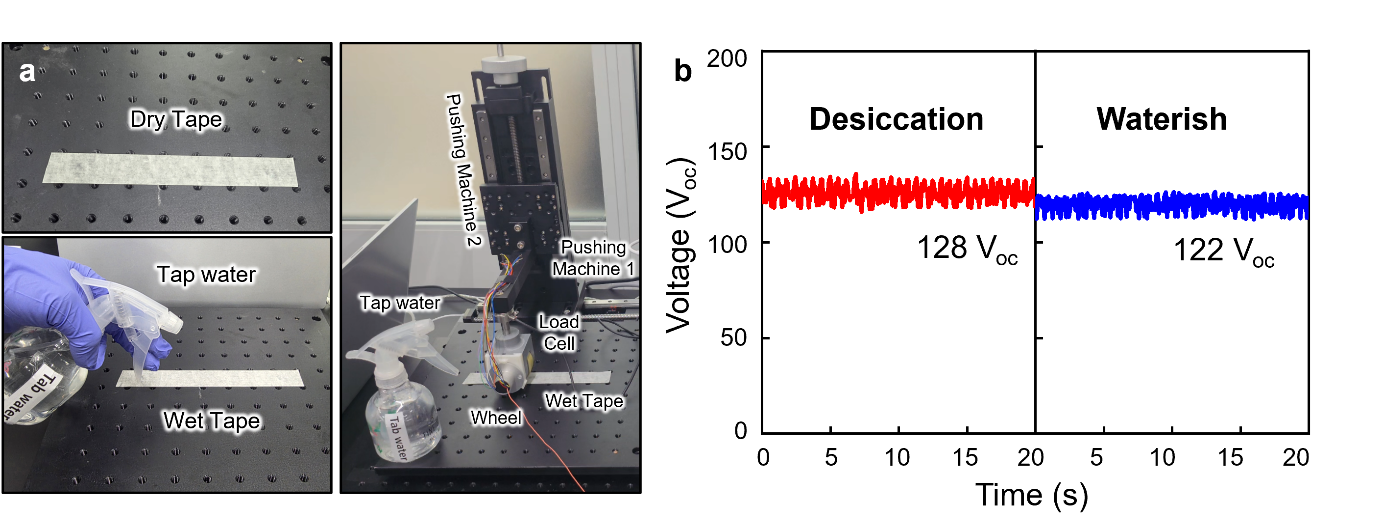
**

**Figure S13. Evaluation of single-electrode DC-PG performance under different ground conditions. a)** Experimental photographs showing the preparation of dry (desiccated) and wet (waterish) ground surfaces using paper tape, and the corresponding measurement setup for output characterization. The wet condition was created by uniformly applying tap water to the tape-covered surface. **b)** Time-dependent open-circuit voltage (V_OC_) signals measured under dry and wet ground conditions.


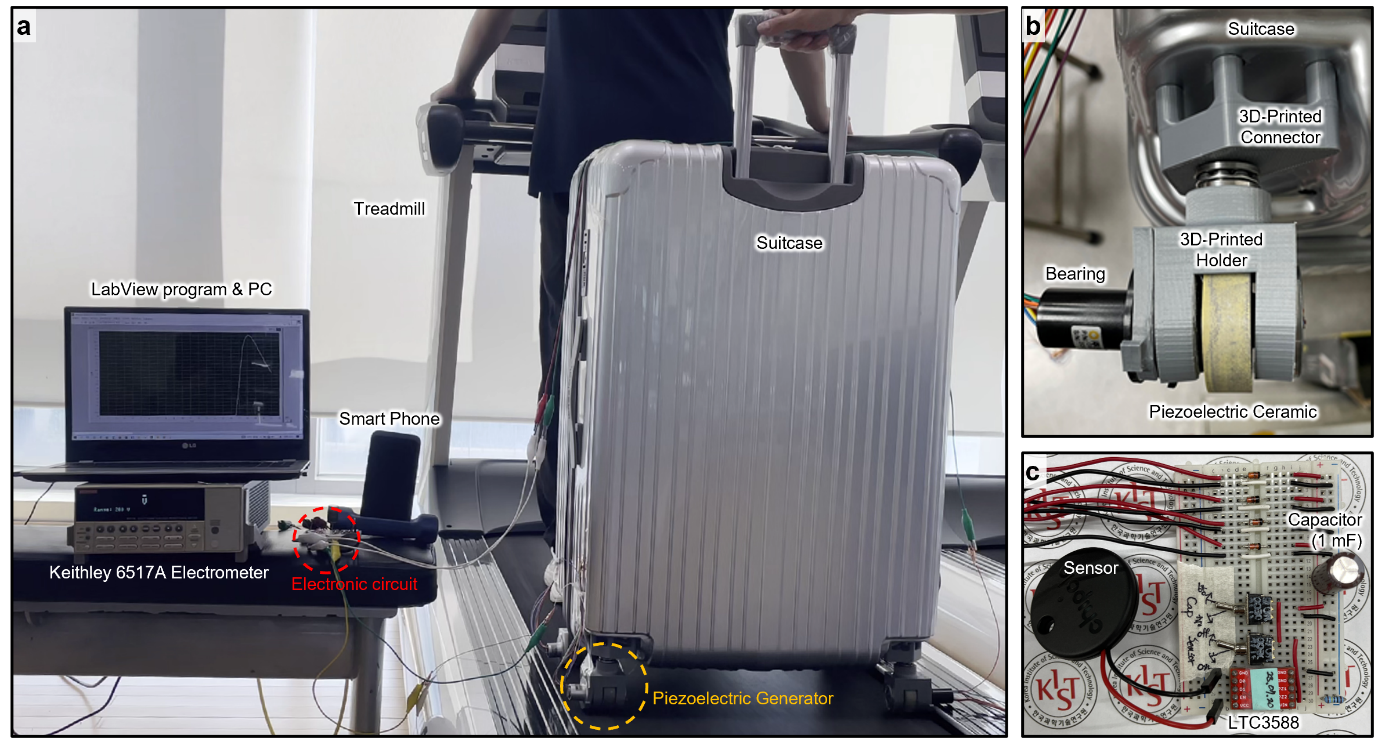


**Figure S14.** **Wheel-shaped DC-PG for powering a location-tracking sensor. A)** Photograph of the treadmill test setup designed to demonstrate the real-time operation of a suitcase location-tracking sensor powered by an in-wheel DC-PG. **B)** Custom-designed 3D-printed wheel housing structure, developed to replace standard suitcase wheels, incorporating wheel-shaped piezoelectric ceramics. **C)** Power conditioning circuitry for sensor operation. Notably, unlike conventional AC generators, no AC-DC conversion stage is required owing to the inherent DC output of the generator.


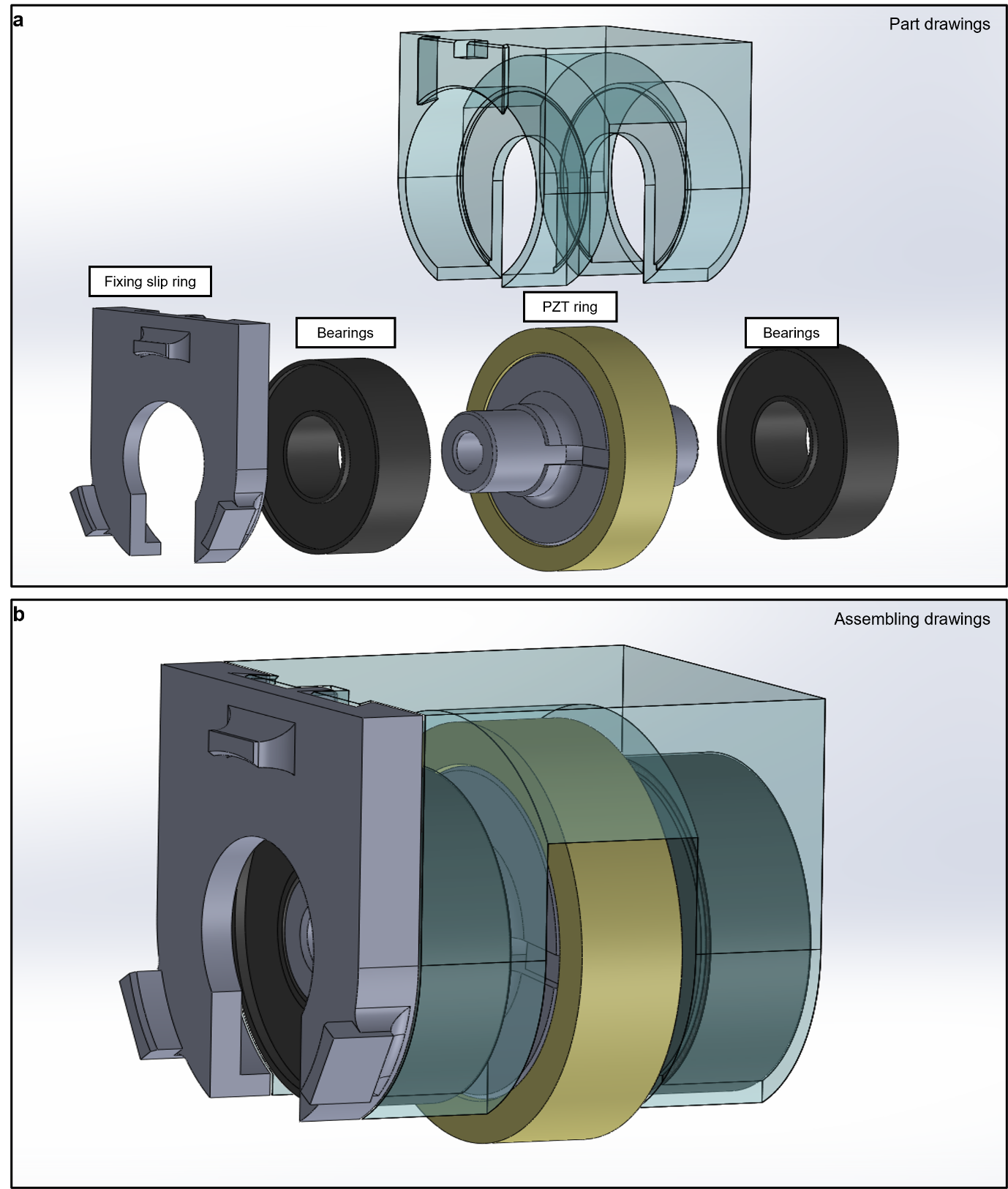


**Figure S15. Structure of the wheel holder designed using SolidWorks 3D CAD. A)** Each individual component was printed separately and subsequently assembled in conjunction with the piezoelectric ring, bearing, and slip ring. **B)** This assembly was then utilized to replace the original wheel of the suitcase.


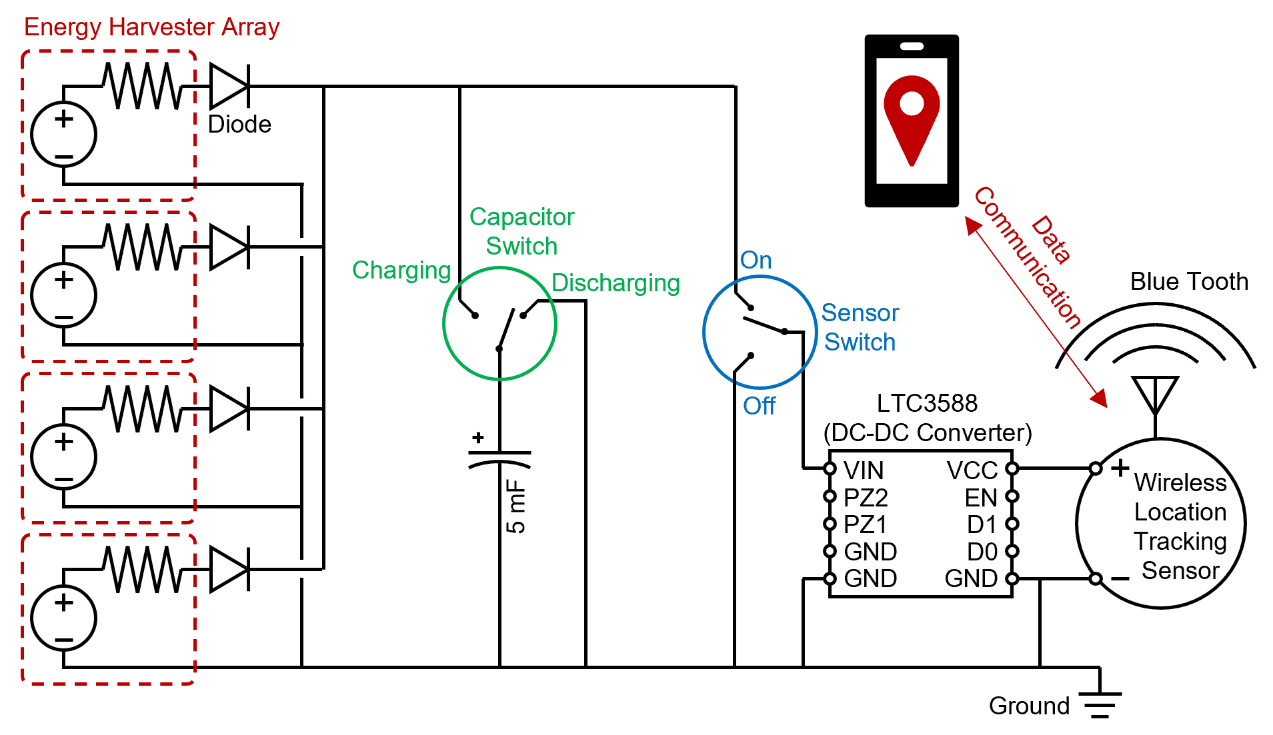


**Figure S16. Electronic schematic of the real-time wireless location-tracking system employing the wheel-shaped DC-PG array.** Owing to the inherently stable DC characteristics, there is no necessity for an AC-DC converter. Solely a DC-DC converter was utilized to supply a consistent voltage of 3V to the sensor.


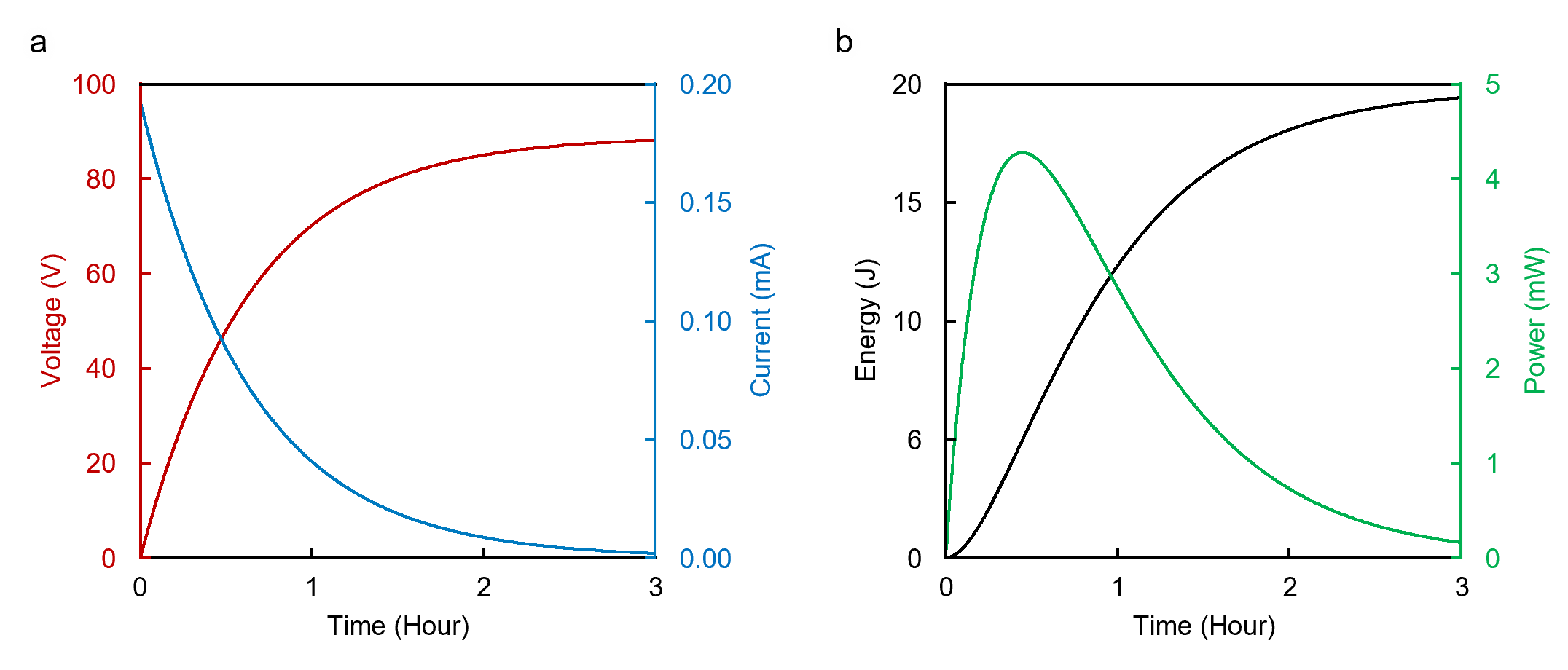


**Figure S17. Long-term capacitor charging test using four-wheel DC generators on a treadmill.** A 5 mF capacitor was employed for the evaluation. **a)** Output voltage and current profiles. **b)** Stored energy in the capacitor and corresponding charging power.


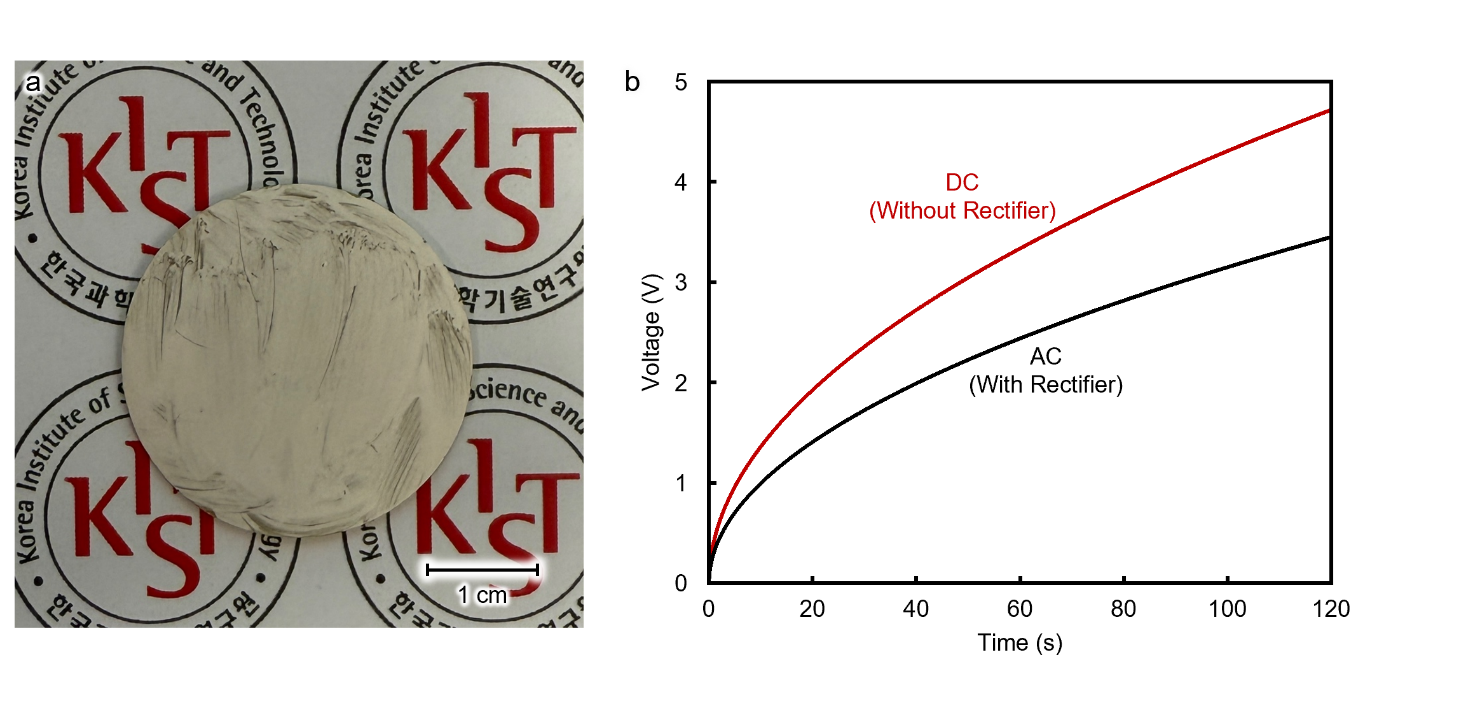


**Figure S18. Performance comparison between AC and DC generators. a)** AC piezoelectric generator with the same volume as the piezoelectric DC generator. **b)** Charging data for a 5 mF capacitor. The AC generator exhibits lower power generation and reduced charging efficiency owing to energy losses during the rectification process.

**3. Supplementary Tables**

**Table S1**. Young’s Modulus Variation based on Floor Material Composition

| Material | Soft rubber | Hard rubber  (Treadmill) | PVC | Tin (Sn) alloy | Aluminum  (A6061) |
| --- | --- | --- | --- | --- | --- |
| Young’s modulus (GPa) | 0.01 | 0.06 | 3.275 | 41.6 | 68.9 |

**4. Supplementary Videos**

**Video S1.** Demonstration of custom-built measurement system and real-time operation used for benchmarking the performance of the wheel-shaped DC-PG.

**Video S2.** Demonstration video showing the application employing a real-time wireless location-tracking system in conjunction with a quad-array of wheel-shaped DC-PG units.

**5. Supplementary Refferences**

[1] R. Bechmann, *Phys. Rev.* **1958**, *110*, 1060.

[2] M. Acosta, N. Novak, V. Rojas, S. Patel, R. Vaish, J. Koruza, G. A. Rossetti Jr., J. Rödel, *Applied Physics Reviews* **2017**, *4*, 041305.

[3] J. Gao, D. Xue, W. Liu, C. Zhou, X. Ren, *Actuators* **2017**, *6*, 24.

[4] J. Shi, M. B. Starr, X. Wang, *Advanced Materials* **2012**, *24*, 4683.
